# An SNX10-dependent mechanism downregulates fusion between mature osteoclasts

**DOI:** 10.1242/jcs.254979

**Published:** 2021-05-11

**Authors:** Maayan Barnea-Zohar, Sabina E. Winograd-Katz, Moran Shalev, Esther Arman, Nina Reuven, Lee Roth, Ofra Golani, Merle Stein, Fadi Thalji, Moien Kanaan, Jan Tuckermann, Benjamin Geiger, Ari Elson

**Affiliations:** 1 Department of Molecular Genetics, The Weizmann Institute of Science, Rehovot 76100, Israel; 2 Department of Immunology, The Weizmann Institute of Science, Rehovot 76100, Israel; 3 Department of Life Sciences Core Facilities, The Weizmann Institute of Science, Rehovot 76100, Israel; 4 Department of Biology, Institute of Comparative Molecular Endocrinology, University of Ulm, 89081 Ulm, Germany; 5 Department of Orthopedics, Istishari Arab Hospital, Ramallah, Palestine; 6 Hereditary Research Laboratory and Department of Life Sciences, Bethlehem University, Bethlehem 0045866, Palestine

**Keywords:** Osteopetrosis, Osteoclast, Cell fusion, SNX10, Bone

## Abstract

Homozygosity for the R51Q mutation in sorting nexin 10 (SNX10) inactivates osteoclasts (OCLs) and induces autosomal recessive osteopetrosis in humans and in mice. We show here that the fusion of wild-type murine monocytes to form OCLs is highly regulated, and that its extent is limited by blocking fusion between mature OCLs. In contrast, monocytes from homozygous R51Q SNX10 mice fuse uncontrollably, forming giant dysfunctional OCLs that can become 10- to 100-fold larger than their wild-type counterparts. Furthermore, mutant OCLs display reduced endocytotic activity, suggesting that their deregulated fusion is due to alterations in membrane homeostasis caused by loss of SNX10 function. This is supported by the finding that the R51Q SNX10 protein is unstable and exhibits altered lipid-binding properties, and is consistent with a key role for SNX10 in vesicular trafficking. We propose that OCL size and functionality are regulated by a cell-autonomous SNX10-dependent mechanism that downregulates fusion between mature OCLs. The R51Q mutation abolishes this regulatory activity, leading to excessive fusion, loss of bone resorption capacity and, consequently, to an osteopetrotic phenotype *in vivo*.

This article has an associated First Person interview with the joint first authors of the paper.

## Abstract (precis)

**Summary:** Fusion of monocytes to become bone-resorbing osteoclasts is limited by an SNX10-dependent cell-autonomous mechanism. Loss of SNX10 function deregulates fusion and generates giant inactive osteoclasts.

## INTRODUCTION

Bone growth and remodeling requires coordinated regulation of osteoblasts, which deposit new bone matrix, and osteoclasts (OCLs) that degrade it. Disrupting the fine balance between these two cellular activities can lead to severe pathological states, such as osteoporosis and osteopetrosis (Del Fattore et al., 2012; Palagano et al., 2018). OCLs are large multinucleated cells that are generated via fusion of monocytic precursor cells following stimulation with M-CSF (also known as CSF-1) and RANKL (also known as TNFSF11) (Teitelbaum, 2007). OCLs adhere to mineralized tissue via integrin-based actin-rich adhesion structures, namely podosomes (Blair et al., 2009; Nakamura et al., 2007; Rodan and Rodan, 1997), that assemble into a robust sealing zone belt (SZ) that confines the bone surface area that is being degraded. The cells then secrete proteases and protons from a specialized region of their ventral membrane known as the ruffled border, thereby degrading the protein and mineral components of the underlying bone (Novack and Teitelbaum, 2008).

An early step in osteoclastogenesis is the fusion of two mononucleated monocytes, which is followed by successive rounds of fusion, mainly with neighboring mononucleated cells (Levaot et al., 2015; Soe et al., 2015) but also with nearby multinucleated OCLs (Jansen et al., 2012). The cells then undergo a maturation process that is manifested, in part, by the assembly of a ventral ruffled border and a robust podosome-based SZ. These changes, together with polarized trafficking of secretory vesicles, enable the mature multinucleated cells to degrade the mineralized bone matrix (Feng and Teitelbaum, 2013; Itzstein et al., 2011; Teitelbaum, 2011).

The formation of multi-nucleated OCLs is based on a general membrane hemifusion mechanism, whereby two cells enter into a tightly docked state in which the outer leaflets of their membranes fuse locally. This is followed by fusion of the inner leaflets and expansion of the resulting pore that connects the cytoplasm of the two cells (Brukman et al., 2019; Chernomordik and Kozlov, 2008; Helming and Gordon, 2009; Martens and McMahon, 2008; Oren-Suissa and Podbilewicz, 2007; Willkomm and Bloch, 2015). This process is mediated by specific fusogens, membranal molecules that act locally on the adjacent cells and promote their fusion (Oren-Suissa and Podbilewicz, 2007). Several molecules, including DC-STAMP (Chiu and Ritchlin, 2016) and OC-STAMP (Witwicka et al., 2015), are required for OCL fusion, but the identity of the actual ‘OCL fusogen(s)’ is still unclear.

Additional functional insights into osteoclastogenesis are provided by specific pathological states in which OCL activity is either excessive or deficient. One such example is autosomal recessive osteopetrosis (ARO), a rare and often lethal genetic disorder that is caused by the failure of OCL-mediated bone resorption (Palagano et al., 2018; Sobacchi et al., 2013). In most ARO cases, OCLs are present but are non-functional (‘OCL-rich ARO’), whereas in other cases OCLs are not formed at all (‘OCL-poor ARO’) (Del Fattore et al., 2008; Palagano et al., 2018; Sobacchi et al., 2013). The typical symptoms of ARO include severe early-onset osteopetrosis, increased bone fragility, impaired development, bone marrow failure and anemia, as well as extramedullary hematopoiesis and absent or impacted teeth (Palagano et al., 2018; Sobacchi et al., 2013). ‘OCL-rich’ ARO was described in 2012 in patients from Palestinian families who were homozygous for an arginine-to-glutamine mutation at position 51 (R51Q) in Sorting Nexin 10 (SNX10) (Aker et al., 2012); it is currently estimated that ∼5% of all ARO cases are SNX10 related (Palagano et al., 2018; Pangrazio et al., 2013; Stattin et al., 2017). SNX10 belongs to a family of over 30 related proteins that participate in regulating endosome sorting and vesicular trafficking. All SNX proteins contain a phox-homology (PX) domain that binds phosphoinositides in endosomal membranes and other subcellular compartments (Cullen, 2008; Teasdale and Collins, 2012; Worby and Dixon, 2002), yet their exact biochemical functions are largely unknown. In OCLs, SNX10 was reported to be associated with the endoplasmic reticulum, with early endosomes, and with the nucleus (Battaglino et al., 2019; Zhu et al., 2012), and to be required for the differentiation of RAW 264.7 cells into OCL-like cells (Zhou et al., 2017; Zhu et al., 2012). It was further shown that mice in which SNX10 was knocked down exhibit massive osteopetrosis due to loss of OCL-mediated bone resorption (Ye et al., 2015).

In order to examine the cellular and whole-organism manifestations of the R51Q mutation in SNX10, we recently generated knock-in mice bearing this mutation (Stein et al., 2020). Homozygous R51Q SNX10 (RQ/RQ) mice exhibit massive early-onset osteopetrosis and display many other clinical symptoms of the corresponding human ARO patients. It was further shown that OCLs of RQ/RQ mice do not resorb bone, *in vivo* and *ex vivo*, due to the absence of ruffled borders and to their inability to secrete protons and acidify the resorption lacunae (Stein et al., 2020). In this study, we address the cell-autonomous manifestations of the homozygous R51Q SNX10 mutation in monocytes throughout their differentiation into OCLs in culture. We show that the mutation results in highly deregulated cell fusion that generates giant cells that fuse continuously and fail to resorb bone, unlike the fusion of wild-type OCLs, which is regulated by a cell-autonomous mechanism. These properties of the mutant OCLs are attributed to loss of function of SNX10 that is caused by the instability of the R51Q SNX10 protein and by its altered functional abilities, which affect membrane trafficking in the mutant cells and lead to loss of their bone-resorbing capacity.

## RESULTS

### Cultured RQ/RQ OCLs are gigantic and unstable

In order to monitor osteoclastogenesis of homozygous R51Q SNX10 (RQ/RQ) OCLs, splenocytes from RQ/RQ and wild-type (+/+) mice were induced to differentiate in culture for 5 to 7 days in the presence of M-CSF and RANKL. Cells were then either fixed and fluorescently labeled for actin, tubulin and DNA, or were subjected to live-cell video microscopy using phase contrast or interference reflection microscopy optics. Splenocytes were used for these experiments as the massive osteopetrosis of RQ/RQ mice radically reduced the bone cavity volume and prevented isolation of sufficient bone marrow cells for routine OCL preparation (Stein et al., 2020).

**Fig. 1.**
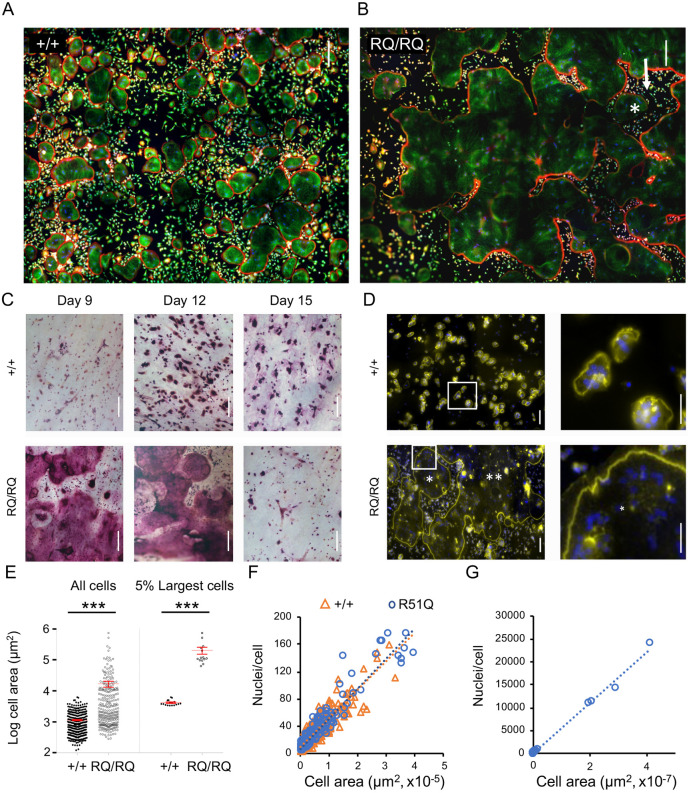
**R51Q SNX10 OCLs are gigantic.** (A,B) Spleen-derived OCLs from +/+ (A) and RQ/RQ (B) mice grown on glass coverslips. Each image is a composite of 12 smaller fields; most of the RQ/RQ image is occupied by part of a single OCL. Cells were stained for actin (red), tubulin (green) and DNA (blue). Boundaries of individual OCLs are marked in Fig. S1C,D. Arrow in B indicates external area that is completely surrounded by the cell, and which contains an OCL (asterisk). (C) Spleen-derived OCLs from +/+ and RQ/RQ mice were grown on bone for the indicated periods, followed by TRAP staining. Figures represent four +/+ and nine RQ/RQ mice from three experiments. RQ/RQ image at day 12 is a composite of two identical images captured at different focal planes. (D) Cells grown on bone were stained for actin (yellow, SZs) and for DNA (blue, nuclei). Most of the RQ/RQ image is occupied by two giant cells that are marked with one or two asterisks, respectively. Each figure is a composite of 15 smaller fields. Right panels show magnified views of boxed areas in the left panels; asterisk (RQ/RQ image) indicates a region within a single OCL. Figures represent three mice/genotype analyzed in two experiments. (E) Size distribution of spleen-derived OCLs grown on bone and stained for TRAP. The left plot shows the average size (mean±s.d.) of multinucleated (≥three nuclei) OCLs shown is 1151±54 µm^2^ (+/+; *N*=353 cells) versus 16,892±3682 µm^2^ (RQ/RQ; *N*=280 cells). The right plot shows the average sizes of the largest 5% of OCLs were 4207±217 µm^2^ (+/+; *N*=17 cells) versus 206,175±52,323 µm^2^ (RQ/RQ; *N*=14 cells). ***P<0.0001 (unpaired two-tailed Student's *t*-test). The *y*-axis scale is logarithmic. Data are from one mouse per genotype, and represent four similar experiments with cells grown on bone or plastic. (F) Scatter diagram depicting the number of nuclei and area per cell in RQ/RQ and +/+ OCLs (*N*=205, 544 cells, respectively) of similar size ranges. Linear trendlines for both genotypes overlap (R^2^: +/+=0.8143, RQ/RQ=0.9095). Data are from one +/+ mouse and two RQ/RQ mice. (G) Similar to F, showing RQ/RQ OCLs of all size ranges. *N*=219 RQ/RQ OCLs, R^2^=0.9951. The large group of cells at the lower left is the same one shown in F. In panels D-G, cells were analyzed at their prime (day 8 for RQ/RQ OCLs, and day 12 for +/+ OCLs, owing to the faster development of the mutant cells). Scale bars: 200 µm (A-C); 100 µm (D, left panels); 50 µm (D, right panels).

Mature +/+ OCLs, cultured on either glass or plastic substrates, were typically round with a projected diameter of 150-300 µm, and displayed a single circumferential SZ-like (SZL) belt of podosomes (Fig. 1A; individual OCL boundaries are marked in Fig. S1A-D). Mononucleated cells and cells in intermediate stages of osteoclastogenesis were scattered between the larger OCLs. Cultures of RQ/RQ OCLs prepared in a similar manner displayed a unique population of extremely large OCLs, each with a projected area of several mm^2^. A typical cell of this category, shown in Fig. 1B and Fig. S1, occupied most of the image space (an area of ∼4.5 mm^2^), and extended significantly beyond the field of view. As in +/+ OCLs, SZLs were noted in RQ/RQ OCLs mainly at the cell periphery. Similarly, large cells were also produced when small numbers of RQ/RQ bone marrow cells were differentiated in culture (Fig. S1E), indicating that production of the giant mutant OCLs is not limited to cells of splenic origin. Large RQ/RQ OCLs often contained ‘islands’ of extracellular space within their projected area that were surrounded by SZLs and typically contained ‘trapped’ mononucleated cells and small multinucleated OCLs (Fig. 1B; Fig. S1D). The origin of these enclosed areas is discussed below.

Importantly, RQ/RQ splenocytes cultured on a physiological bone substrate also fused into huge multi-nucleated cells (Fig. 1C). These large RQ/RQ OCLs stained positively for tartrate-resistant acid phosphatase (TRAP, encoded by *ACP5*), but the staining was weak and diffuse compared to +/+ cells. Longer-term studies indicated that the lifespan of RQ/RQ OCLs was significantly shorter than their +/+ counterparts (Fig. 1C, day 15). Each RQ/RQ OCL grown on bone displayed a single peripheral SZ, in contrast to +/+ cells that typically contained several much smaller SZs per cell (Fig. 1D).

Examination of individual OCLs indicated that the average area of RQ/RQ OCLs was 14.6× larger than +/+ OCLs (Fig. 1E, left); this ratio increased to 49 when the upper five percentiles of the two cell populations were compared (Fig. 1E, right). In order to examine whether the large size of RQ/RQ OCLs results from enhanced cell spreading or from increased fusion, we plotted the number of nuclei in individual +/+ and RQ/RQ OCLs as a function of their area. As shown in Fig. 1F, the projected area and number of nuclei were highly correlated in both RQ/RQ and +/+ OCLs, including in the exceptionally large RQ/RQ OCLs (e.g. Fig. 1G). We conclude that the large projected area of the giant RQ/RQ OCLs is attributable to the increased fusion of mutant cells.

### RQ/RQ OCLs exhibit altered fusion dynamics

**Fig. 2.**
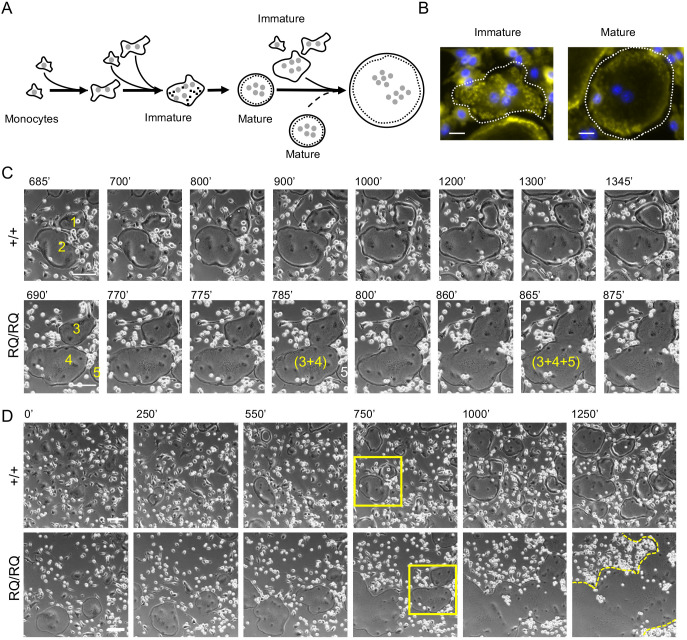
**RQ/RQ OCLs are large due to deregulated fusion.** (A) Schematic outline of osteoclastogenesis as monocytes develop into OCLs when cultured with M-CSF and RANKL, based on live cell imaging studies. Small shaded circles, nuclei; black dots, podosomes; dashed circular lines, the podosomal SZL structure. Dashed arrow highlights fusion between two mature round OCLs, which is detected only in RQ/RQ cultures. (B) Immature (left) and mature (right) +/+ OCLs. Cells were stained for actin (podosomes, yellow) and DNA (nuclei, blue). Dashed white line indicates cell boundaries. (C) Image series showing mature round +/+ OCLs interacting for 660 min without fusing (cells 1 and 2, Movie 1), versus two rapid fusion events between mature RQ/RQ OCLs that occur during 175 min (cells 3, 4 and 5; from Movie 2). Small bright cells are monocytes. Numbers indicate individual cells; parentheses indicate fused cells. (D) Lower magnification images showing fusion of +/+ (from Movie 1) and RQ/RQ (Movie 2) OCLs. Rectangles indicate areas enlarged in C. Dashed line in RQ/RQ culture, t=1250 min, marks outline of a single large OCL. Composite movies of larger field views of OCL fusion are presented as Movies 3 (+/+) and 4 (RQ/RQ). Scale bars: 20 µm (B); 100 µm (C,D).

The fusion dynamics of +/+ and RQ/RQ OCLs were compared by analyzing phase-contrast live cell movies (Movies 1-4; Fig. 2) in conjunction with immunofluorescence microscopy of fixed cells. As noted previously (Levaot et al., 2015; Soe et al., 2015), RANKL induced +/+ monocytes to fuse, and the resulting binucleated cells grew further by fusing with adjacent mononucleated cells (Fig. 2A) and, occasionally, with neighboring OCLs. Many of these initially formed multinucleated cells were motile, assumed an irregular shape and displayed podosomes scattered throughout their ventral membrane (immature cell, Fig. 2B). With time, these cells assumed a rounder and smoother shape, which we refer to as ‘mature’ morphology. Concomitantly, these cells became less motile and developed a podosomal belt at their periphery (mature cell, Fig. 2B). Mature +/+ OCLs continued to grow, fusing either with mononucleated cells or with neighboring immature multinucleated cells (Fig. 2A; Movies 1, 3). Fusion between pairs of mature +/+ OCLs was, however, observed very rarely. Most pairs of mature +/+ OCLs remained juxtaposed for long periods of time and then separated (e.g. cells 1 and 2 in Fig. 2C). In agreement with this observation, the sequence of fusion events of +/+ OCLs often ended when mature cells became completely surrounded by other mature OCLs with which they did not fuse (Fig. 2D; Fig. S1F, Movie 3). RQ/RQ OCLs displayed a similar morphological maturation process. Yet, in sharp contrast with +/+ OCLs, mature RQ/RQ OCLs readily fused with each other (dashed arrow in Fig. 2A), forming giant OCLs that continued to fuse and grow ever larger (Movies 2, 4; cells 3, 4 and 5 in Fig 2C,D). Fusion of the mutant OCLs ended only when no fusion partners remained. Fusion between mutant OCLs typically started at several points along the cell-cell interface and proceeded rapidly, often engulfing areas of extracellular space that remained surrounded by podosomal SZLs (Fig. 1B; Fig. S1D). These areas were gradually integrated into the surrounding cell and their SZLs dissolved. Taken together, these observations point to the existence of a regulatory mechanism that blocks fusion between mature +/+ OCLs, and indicates that this mechanism is dependent on SNX10 and that it is disabled in RQ/RQ OCLs.

**Fig. 3.**
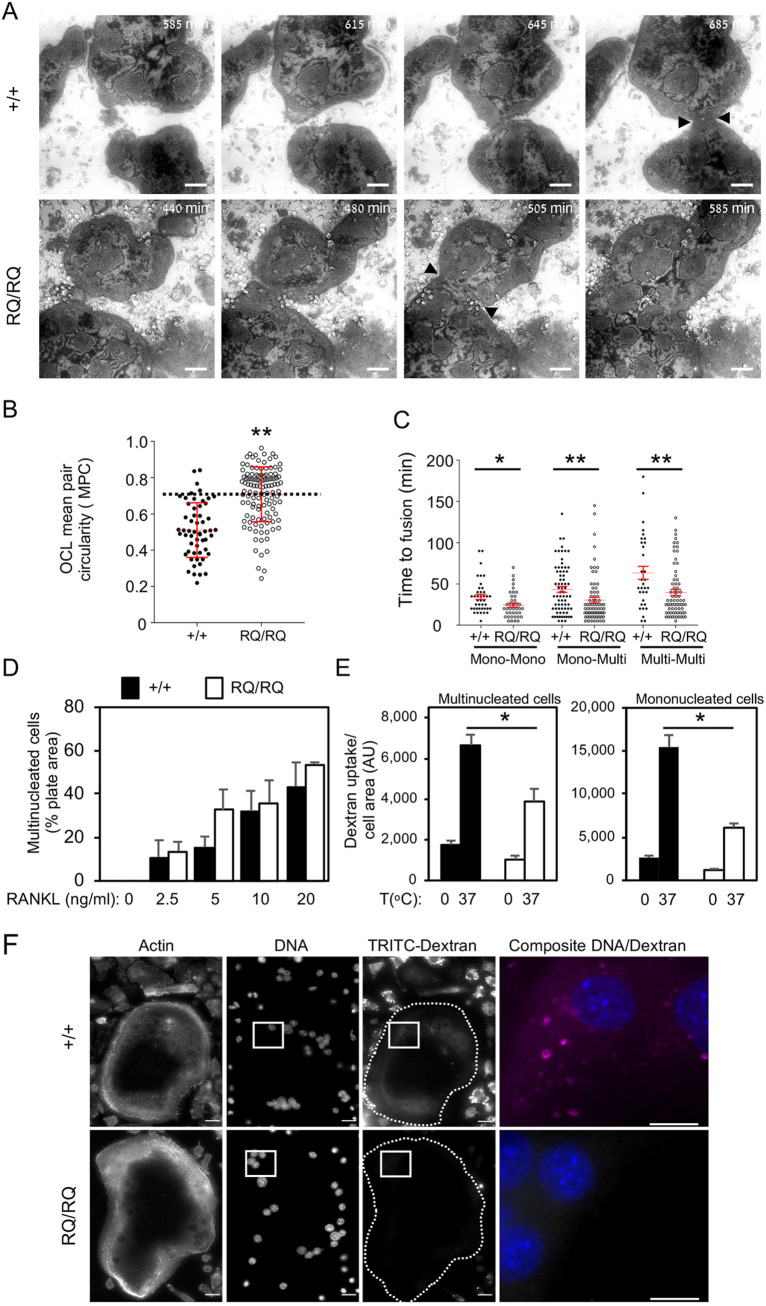
**Fusion dynamics of homozygous R51Q SNX10 OCLs.** (A) Images captured from IRM movies showing fusion of +/+ and RQ/RQ OCLs at the indicated times (from Movies 5 and 6, respectively). Arrowheads mark the area of contact between +/+ cells (top, t=685 min) and fusion between RQ/RQ cells (bottom, t=505 min). See also Figs S2, S3. (B) Scatter plot showing the MPC of pairs of multinucleated +/+ and RQ/RQ OCLs that fused, from Movies 3 and 4. Horizontal dashed line indicates MPC=0.7. MPC values (mean±s.d.) are 0.511±0.150 (+/+) and 0.708±0.151 (RQ/RQ), *N*=56 and 117 cell pairs, respectively. **P<0.0001 (unpaired two-tailed Student's *t*-test). (C) Elapsed time between initial cell-cell contact and subsequent fusion. Mono, mononucleated monocytes; Multi, multinucleated OCLs. Data are mean±s.e., *N*=31-73 fusion events per category and genotype. Data are from *N*=3 +/+ and 3 RQ/RQ mice from three experiments. *P=0.017, **P<0.0085 (unpaired two-tailed Student's *t*-test). (D) Percentage of the well surface area covered by +/+ and RQ/RQ OCLs differentiated with RANKL for 5 days (mean±s.e., *N*=3 mice/genotype in three experiments). OCL images are presented in Fig. S4A. (E) Uptake of dextran by +/+ and RQ OCLs (left) and mononucleated cells (right) following incubation for 30 min at 0 or 37°C in the presence of TRITC-dextran (40 kDa). Fluorescence images were acquired at identical settings for all conditions. Shown is the total TRITC fluorescence per cell normalized by cell area (mean±s.e.): *P≤0.0002 by one-way ANOVA with Tukey-Kramer post-hoc analysis. *N*=29-32 (+/+ multinucleated) and 11-13 (RQ/RQ multinucleated) or 59-64 (+/+ mononucleated) and 39-56 (RQ/RQ mononucleated) cells/bar from two experiments. Dextran uptake at 0°C and cell size ranges were similar in both genotypes. (F) Images of mature +/+ and RQ/RQ OCLs that had been incubated with TRITC-dextran for 30 min at 37°C. Dashed lines indicate cell boundaries. Scale bars: 100 µm (A); 20 µm (F); 10 µm (F, composite image at the far right). AU, arbitrary units.

Complementary information about differential osteoclastogenesis in RQ/RQ OCLs and their +/+ counterparts was obtained by dynamic live cell IRM, a microscopy-based imaging method that enables direct examination in culture of the gap between the ventral membrane of live adherent cells and the underlying substrate. In IRM images, dark regions indicate a closer local proximity of the ventral membrane to the substrate (Movies 5, 6; Fig. 3A; Figs S2, S3). When examined by IRM, large +/+ OCLs exhibited complex and rapidly changing shading patterns, indicating that the distance between the cells and the underlying surface is highly dynamic. The peripheral regions of these cells, in which SZLs are located, were typically darker and more stable, consistent with the presence of stable adhesions in these areas. This region remained dark also in mature juxtaposed +/+ OCLs that did not fuse (Fig. 3A, +/+ cells, t=685 min). In contrast, the local IRM intensity of the SZLs of neighboring RQ/RQ OCLs became considerably lighter just before and during the fusion event (Fig. 3A, RQ/RQ cells, t=505 min), indicating that local adhesion to the substrate is reduced during fusion between mature RQ/RQ OCLs.

### Quantification of the morphological ‘signature’ of mature OCLs

The observations described above indicate that mature +/+ and RQ/RQ OCLs differ radically in their capacity to fuse with other mature OCLs. As described above, maturation of OCLs grown on non-degradable surfaces includes three main manifestations: (1) loss of the ability to fuse with other mature OCLs; (2) the formation of a single peripheral SZL; and (3) a circular morphology. In order to quantify the morphological manifestations of the mature state in cultured OCLs of both genotypes, we employed comparative morphometry. Specifically, by running osteoclastogenesis phase-contrast movies ‘backwards’, we identified pairs of fusing multinucleated OCLs in +/+ and RQ/RQ cultures and marked their individual boundaries just before they fused. We then calculated the circularity of each of these cells {4π[(cell area)/(cell perimeter)^2^]}, where a value of 1 indicates a perfect circle. As fusion occurs between cell pairs, we represented each pair of fusing OCLs by the arithmetic mean of their individual circularity values (mean pair circularity, MPC). Low-MPC fusion events between multinucleated cells, in which one or both were immature and irregularly shaped, occurred in both genotypes (Fig. 3B). However, high-MPC fusion events between pairs of mature round OCLs were significantly more prevalent in RQ/RQ cultures than in +/+ cultures. For example, an MPC value of 0.7 is close to the median of RQ/RQ fusion events, but represents the 90th percentile of +/+ fusions (Fig. 3B). High-MPC interactions between +/+ OCLs were common; however, most ended in separation after a relatively long time (393.8±56.2 min, mean±s.e., *N*=59 interactions), indicating that this particular fusion modality is inhibited in this genotype. Collectively, these results agree well with the observation that fusion between pairs of round morphologically mature OCLs is much more common among RQ/RQ OCLs than among +/+ OCLs.

Further studies revealed that in +/+ cultures, fusion events involving two mononucleated cells or a mononucleated cell and a multinucleated cell were rapid, with the cells juxtaposing their membranes and fusing after 30-60 min (Fig. 3C, mono-mono and mono-multi modalities, +/+ cells). Fusion between two multinucleated cells, which in +/+ cultures refers predominantly to pairs of OCLs with low MPC values, was slower than other fusion modalities (Fig. 3C, multi-multi +/+ cells) and, as indicated, was a rare event. In contrast, fusion between RQ/RQ cells in all three modalities proceeded faster than in +/+ cells (Fig. 3C, RQ/RQ cells). As indicated, all cases in which multinucleated RQ/RQ cells became adjacent to each other led to their rapid fusion, irrespective of their MPC values (Movies 2, 4), highlighting a major functional distinction between OCLs of the two genotypes.

### Deregulated fusion of RQ/RQ OCLs is not caused by aberrant RANKL signaling

Cell fusion during osteoclastogenesis is driven by the continuous presence of RANKL; hence, we examined whether the deregulated fusion of RQ/RQ OCLs might be caused by increased sensitivity to this cytokine. Similar to +/+ cells, RQ/RQ OCL precursor cells neither differentiated nor fused in the absence of RANKL (Fig. 3D; Fig. S4A), indicating that the production of RQ/RQ OCLs is strictly RANKL dependent. Moreover, when +/+ and RQ/RQ cells were cultured separately in the presence of increasing concentrations of RANKL, the relative area of the growth surface covered by multinucleated OCLs increased in a dose-dependent manner that was similar in both genotypes (Fig. 3D). Yet, individual RQ/RQ OCLs were fewer and consistently larger than the more numerous +/+ OCLs grown at the same concentration of RANKL (Fig. S4A), indicating that RQ/RQ OCLs fuse abnormally also at low concentrations of RANKL. qPCR studies showed that mRNAs for RANKL-induced proteins, including the key osteoclastogenic transcription factor NFATc1, TRAP, cathepsin K, the ATP6V1D2 V-ATPase subunit, CLCN7, the RANKL receptor RANK, and the fusion-related proteins DC-STAMP and OC-STAMP, were expressed at similar levels in +/+ and in RQ/RQ OCLs (Fig. S4B). We conclude that +/+ and RQ/RQ OCLs and their precursor cells respond similarly to RANKL, and that the fusion phenotype of the mutant cells is not caused by abnormal sensitivity to this cytokine.

### RQ/RQ OCLs exhibit reduced dextran endocytosis

SNX10 participates in vesicle trafficking and in endocytosis, cellular activities that are critical for osteoclastogenesis and the disruption of which may lead to major membrane-related phenotypes, such as loss of ruffled border structures and failure of acidification and bone resorption by OCLs (Palagano et al., 2018; Pangrazio et al., 2013; Stein et al., 2020). Similar defects cause aberrant cell fusion in other cell types (Smurova and Podbilewicz, 2017). In order to directly examine the effect of R51Q SNX10 on endocytosis in OCLs, we measured dextran internalization by these cells, a process that occurs by both fluid-phase and receptor-mediated endocytosis (Pustylnikov et al., 2014). Cells were cultured in the presence of tetramethyl-rhodamine-6-isothiocyanate (TRITC)-labeled dextran, and their ability to internalize the compound was evaluated by fluorescence microscopy. Control OCLs from both genotypes incubated at 0°C exhibited low and similar levels of TRITC signals (Fig. 3E). Internalized dextran was clearly visible in mononucleated and immature polynucleated cells of both genotypes that were incubated at 37°C (Fig. 3F, TRITC-dextran panels). In mature +/+ OCLs dextran was clearly internalized, manifested by a strong punctate cytoplasmic labeling; dextran uptake by mature RQ/RQ OCLs was reduced by 40.8% (Fig. 3E,F). Dextran uptake into mononucleated RQ/RQ cells was also reduced relative to +/+ controls (Fig. 3E).

### The R51Q SNX10 protein is unstable, exhibits aberrant lipid-binding properties and is mislocalized in RQ/RQ OCLs

**Fig. 4.**
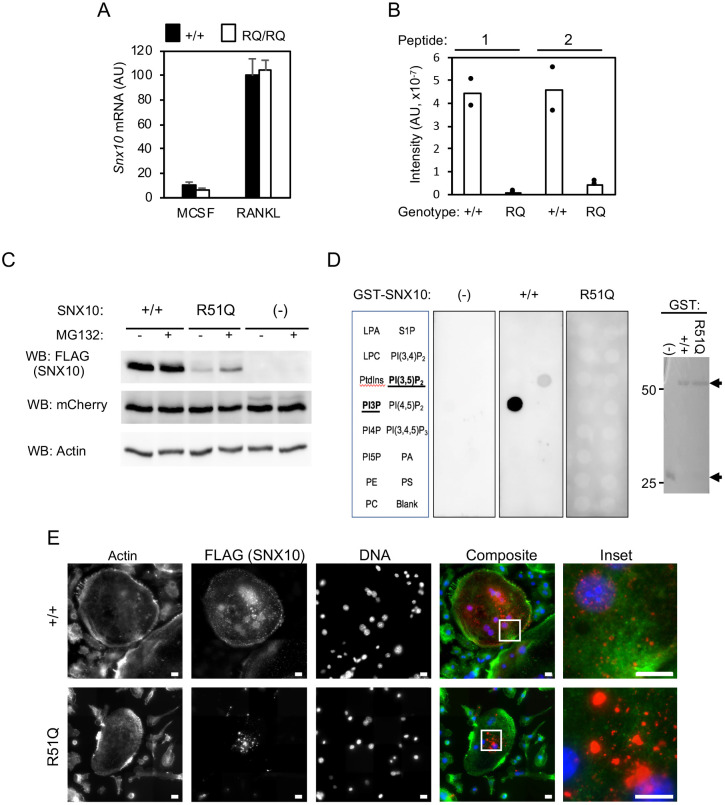
**Mislocalization, reduced stability and reduced lipid-binding ability of R51Q SNX10 in RQ/RQ OCLs.** (A) qPCR analysis of *Snx10* mRNA expression (mean±s.d.) in +/+ and RQ/RQ monocytes/macrophages (M-CSF) or OCLs produced from them (RANKL). *N*=12 +/+ and 14 RQ/RQ mice. (B) Targeted proteomics of +/+ and RQ/RQ OCL lysates. Two peptides (peptide 1, LQSNALLVQLPELPSK; and peptide 2, NLFFNMNNR, starting at residues 64 and 80, respectively, in the SNX10 sequence) were detected and quantified. *N*=2 mice/genotype. (C) RAW264.7 cells were infected with lentiviral vectors expressing FLAG-tagged wild-type (+/+) or R51Q SNX10, or were empty (−). Some cells were treated with 20 nM MG-132 for 4 h before processing. mCherry protein, which is co-expressed from the same lentiviral constructs, and actin serve as infection/expression and loading controls, respectively. (D) In the left panels, equal amounts of GST fusion proteins of +/+ or R51Q SNX10 were used to probe membrane phospholipid arrays. (−), negative control (GST alone). Array incudes lysophosphatidic acid (LPA), lysophosphocholine (LPC), phosphatidylinositol (PtdIns), PtdIns (3) phosphate (PI3P), PtdIns (4) phosphate (PI4P), PtdIns (5) phosphate (PI5P), phosphatidylethanolamine (PE), phosphatidylcholine (PC), sphingosine 1-phosphate (S1P), PtdIns (3,4) bisphosphate [PI(3,4)P_2_], PtdIns (3,5) bisphosphate [PI(3,5)P_2_], PtdIns (4,5) bisphosphate [PI(4,5)P_2_], PtdIns (3,4,5) trisphosphate [PI(3,4,5)P_3_], phosphatidic acid (PA) and phosphatidylserine (PS). The right panel shows a Coomassie-stained protein gel documenting the amounts of GST proteins used to probe the arrays. One experiment out of two performed is shown. Arrows mark GST-SNX10 (top) and GST (bottom). (E) Localization of wild-type and mutant SNX10 in OCLs. cDNA for +/+ SNX10 bearing a C-terminal FLAG tag was expressed in wild-type OCLs by adenoviral transduction; a tagged cDNA for R51Q SNX10 was similarly expressed in RQ/RQ OCLs, and the cells were probed for actin (green fluorescence), FLAG (SNX10, red) and DNA (blue). No FLAG signal was detected in untransduced cells (not shown). Scale bars: 10 µm. AU, arbitrary units.

Next, we examined the expression and functional properties of wild-type and mutant SNX10 in OCLs. qPCR studies indicated that *Snx10* mRNA is induced during OCL differentiation, and that total *Snx10* mRNA levels are similar in +/+ and RQ/RQ OCLs (Fig. 4A). cDNA sequencing confirmed that +/+ OCLs express wild-type *Snx10* mRNA and that RQ/RQ OCLs express mutant mRNA exclusively (Fig. S4C). Detection of endogenous SNX10 protein in OCLs by protein blotting with commercial anti-SNX10 antibodies yielded inconsistent results; hence, we examined SNX10 expression directly using targeted proteomics. Analysis of whole-cell lysates of +/+ OCLs identified two peptides derived from the SNX10 protein. These peptides were either absent or were detected at low levels in RQ/RQ OCLs (Fig. 4B), indicating that the R51Q SNX10 protein is present at low amounts in the mutant OCLs. Poor expression of the R51Q SNX10 protein was also observed when the exogenous mutant protein was expressed in RAW264.7 cells and detected via its FLAG tag (Fig. 4C). Exposing these cells to the proteasome inhibitor MG132 increased detection of R51Q SNX10 protein (Fig. 4C), indicating that its low levels are caused at least in part by its reduced stability and degradation.

Arginine 51 is located within the phosphoinositide-binding PX domain of SNX10, suggesting that the R51Q mutation might affect its lipid-binding specificity and through it the function of the residual mutant protein. In order to examine this possibility, we produced wild-type and R51Q SNX10 proteins in bacteria and used equal amounts of each protein to probe a phospholipid array *in vitro*. As shown in Fig. 4D, wild-type SNX10 bound phosphatidylinositol 3-phosphate (PI3P) and phosphatidylinositol 3,5-bisphosphate [PI(3,5)P_2_], whereas R51Q SNX10 did not bind any of the phospholipids present on the array. As lipid binding by the PX domain enables SNX10 to associate with vesicular membranes, we examined whether the R51Q mutation altered the cellular localization pattern of SNX10. To this end, we expressed FLAG-tagged cDNAs for +/+ SNX10 and for its R51Q mutant in +/+ and in RQ/RQ OCLs, respectively, followed by immunofluorescence staining with anti-FLAG antibodies. +/+ SNX10 was readily expressed in the cells, and appeared as small regularly shaped punctate signals in the cytosol that were consistent with vesicular localization (Fig. 4E). In agreement with its low amounts in OCLs, exogenous R51Q SNX10 protein was expressed at lower levels and in fewer cells, where it gave rise to larger irregularly shaped fluorescent signals that may represent protein aggregates (Fig. 4E). Collectively, these data suggest that the RQ/RQ OCL fusion phenotype is caused by loss of function of SNX10 due to a severe reduction in the amount of R51Q SNX10 protein. This is likely caused by the inability of the R51Q SNX10 protein to bind specific phospholipids, which leads to its subsequent mislocalization and degradation.

### R51Q SNX10 induces OCL hyperfusion by a loss of function mechanism

**Fig. 5.**
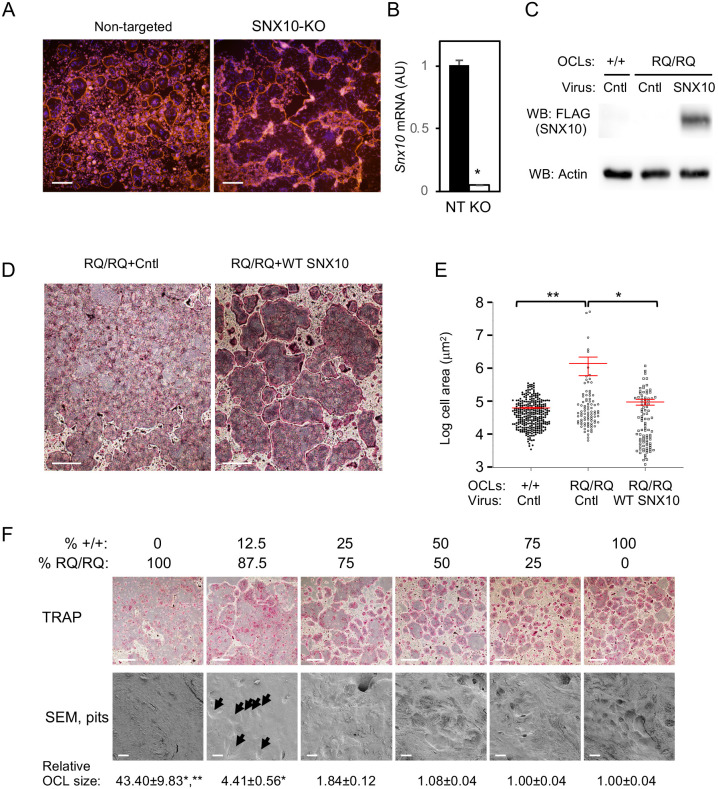
**The R51Q SNX10OCL fusion phenotype is caused by loss of SNX10 function.** (A) SNX10-knockout (SNX10-KO) RAW 264.7 cells and control cells treated with a non-targeting control sgRNA were seeded on plastic plates and differentiated into OCL-like cells with M-CSF and RANKL for 4 days. The cells were stained for actin (TRITC-phalloidin, red) and DNA (Blue). Individual OCL boundaries and the sequence of the mutated locus are presented in Fig. S5. Data are from one SNX10-KO clone and are representative of six independent clones. (B) qPCR analysis of *Snx10* mRNA expression (mean±s.e.) in non-targeted RAW 264.7 cells (NT) and in SNX10-KO (KO) cells shown in A. *P*=0.00049 (unpaired two-tailed Student's *t*-test, *N*=3 repeats). (C) Protein blot documenting expression of exogenous wild-type SNX10 in RQ/RQ OCLs, for panels D and E. Cntl, control adenovirus expressing GFP. SNX10, adenovirus expressing wild-type FLAG-tagged SNX10. (D) Images of TRAP-stained RQ/RQ OCLs infected with control (+Cntl) or wild-type SNX10 (+WT SNX10) adenoviruses. (E) Areas (mean±s.e.) of OCLs infected with control or wild-type SNX10 adenoviruses, from one experiment representative of two performed. *P=0.0042, **P=0.0002 (one-way ANOVA). *N*=88-309 multinucleated cells/category from three experiments**.** The *y*-axis scale is logarithmic. (F) +/+ and RQ/RQ monocytes/macrophages were mixed as indicated and differentiated into OCLs on plastic (TRAP) or bone (SEM). TRAP-stained cells (top) and scanning electron microscopy (SEM) of pits excavated by the cells (bottom) are shown, as well as cell sizes (mean±s.e., relative to the culture of 100% +/+ cells). *P<0.0001 versus 100% +/+ cell culture, **P<0.0001 versus 12.5% +/+ cells (unpaired two-tailed Student's *t*-test). *N*=222-606 cells/category (100%-12.5% +/+ cells) and *N*=28 cells (0% +/+ cells). Arrows mark resorbed pits in the 12.5% SEM image. Images are from one experiment, representing six TRAP and two SEM experiments. Scale bars: 500 µm (A); 250 µm (D,F, TRAP); 20 µm (F, SEM). AU, arbitrary units.

In order to examine directly whether the RQ/RQ OCL hyperfusion phenotype is caused by loss of function of SNX10, we knocked out the *Snx10* gene in RAW 264.7 cells using CRISPR technology (Fig. S5A). Treatment of SNX10-KO RAW 264.7 cells with RANKL induced their differentiation into mature OCL-like cells, which fused continuously with each other and formed gigantic cells that resembled those observed in cultures of primary RQ/RQ OCLs (Fig. 5A; individual OCL boundaries are marked in Fig. S5D,E). In contrast, similar treatment of control unmodified RAW 264.7 cells resulted in the formation of mature OCL-like cells that typically did not fuse with each other and were much smaller than SNX10-KO RAW 264.7 cells (Fig. 5A; Fig. S5B-E). qPCR analysis confirmed that control RANKL-treated RAW 264.7 cells expressed *Snx10* mRNA, whereas the SNX10-KO cells did not (Fig. 5B). Complete loss of SNX10 function by knockout of *Snx10* in differentiating RAW 264.7 cells is therefore sufficient to induce a hyperfusion phenotype.

In related studies, expression of wild-type SNX10 in mutant OCLs blocked the formation of giant OCLs (Fig. 5C-E). Notably, expression of R51Q SNX10 in RQ/RQ OCLs did not prevent the formation of the giant OCLs (Fig. S5F,G), indicating that the mutant SNX10 protein lacks the functional ability to do so. In a complementary experiment, which simulates a bone marrow transplantation-like scenario of ARO patients that often follows limited myeloablative pretreatment, we examined OCL development and activity in mixed cultures of wild-type and mutant cells. For this purpose, +/+ and RQ/RQ monocyte precursor cells were seeded in various proportions, as indicated in Fig. 5F, and then differentiated into OCLs. Separate studies indicated that when grown together, +/+ and RQ/RQ cells fuse to form genetically hybrid heterokaryons (Fig. S6). Cultures comprised entirely of RQ/RQ cells contained very large cells that exhibited no resorbing activity when seeded on bovine bone (Fig. 5F). Adding as little as 12.5% +/+ cells resulted in detectable bone-resorbing activity, and the average size of the resulting multinucleated cells was reduced tenfold. Increasing the fraction of +/+ cells to 25% resulted in close to normal bone-resorbing activity and OCL size, and cultures containing 50% or more +/+ cells were indistinguishable in size and activity from pure +/+ OCL cultures (Fig. 5F). Growth of +/+ or RQ/RQ OCLs in medium conditioned by prior growth of OCLs from the other genotype did not alter the fusion characteristics of either genotype (data not shown), indicating that the RQ/RQ phenotype is not induced by differential secretion of soluble factors from the OCLs. Fusion of even small amounts of +/+ cells with RQ/RQ cells can therefore provide sufficient wild-type SNX10 protein to rescue the fusion and activity phenotypes of the mutant OCLs.

## DISCUSSION

**Fig. 6.**
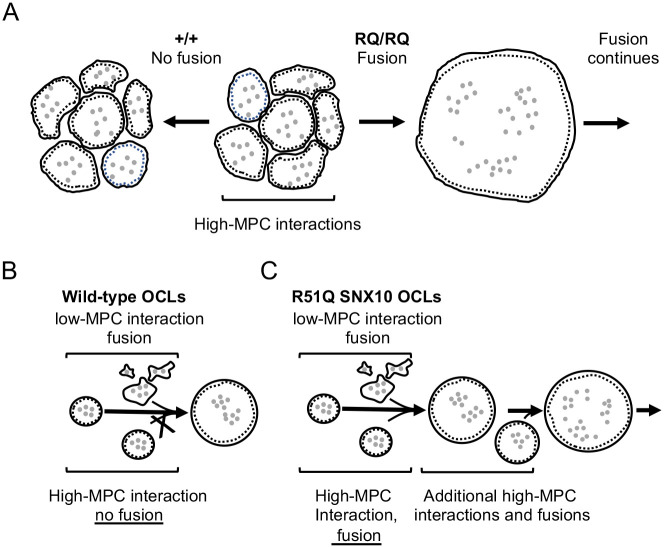
**R51Q SNX10 abolishes a regulatory mechanism that blocks fusion between pairs of mature OCLs.** (A) During osteoclastogenesis *in vitro*, round mature OCLs become surrounded by similarly mature OCLs, with which they form high-MPC interactions. In +/+ cultures (left), fusion halts at this point, but in RQ/RQ cultures (right) this is followed by massive continuous fusion. Gray dots indicate nuclei, and dashed lines represent SZLs. (B) Pairs of mature round OCLs of high-MPC values do not fuse in +/+ cultures, but fuse readily in RQ/RQ cultures. (C) OCL pairs of low-MPC values fuse in both genotypes.

The dynamic process whereby OCL precursor cells fuse to form multinucleated bone-resorbing OCLs has been extensively investigated in recent years (Levaot et al., 2015; Soe et al., 2015). In wild-type cultures, this process yields OCLs with wide, yet predictable, ranges of size and number of nuclei that, in mice, rarely exceed 2×10^5 ^µm^2^ and 100 nuclei, respectively (Fig. 1F). This rather common observation, per se, indicates that osteoclastogenic cell fusion is a tightly regulated process that is blocked when the heterokaryons reach a ‘mature stage’. The existence of an upper limit to OCL size is broadly accepted, yet the nature of the regulatory mechanism that underlies the signal that stops fusion is unknown. This study sheds new light on this process and shows that the membrane trafficking-associated protein SNX10 actively participates in downregulating fusion between mature OCLs (Fig. 6). In particular, the finding that a defined point mutation in a single protein induces cell-autonomous deregulated fusion indicates that OCL size is actively regulated by a cellular-genetic mechanism. The data suggest that this mechanism functions by limiting fusion between pairs of mature OCLs, that SNX10 is essential for this process, and, surprisingly, that no backup mechanism halts fusion between mature OCLs once this mechanism is disrupted.

How is OCL maturation manifested? As OCLs differentiate and fuse, these cells undergo morphological and functional changes that enable them to effectively degrade bone. Here, we show that among these changes, cultured OCLs undergo ‘morphological maturation’ and assume a more circular shape, and concomitantly lose their ability to fuse with other morphologically mature, multinucleated OCLs. Interestingly, although mature OCLs cannot fuse with each other, they readily fuse with less mature cells. This observation highlights an intriguing asymmetry, whereby mature OCLs cannot initiate fusion but can fuse with immature cells that apparently initiate this process. Although the underlying mechanism is unclear, this asymmetry is reminiscent of the initial stages of osteoclastogenesis, in which long-term priming by RANKL differentiates a subpopulation of monocytes into ‘founder cells’, which fuse with ‘follower’ monocytes that are fusion competent but cannot initiate the fusion process (Levaot et al., 2015). Similar asymmetry during cell-cell fusion has also been described during myoblast-myotube fusion in *Drosophila* (Dworak and Sink, 2002), although it is not a general aspect of cell fusion (reviewed by Hernández and Podbilewicz, 2017; Schejter, 2016).

The correlation between the shape of an OCL and its ability to fuse was further examined in a quantitative manner by calculating the circularity of the cell, a morphological parameter that is independent of object size. As cell fusion occurs between pairs of interacting cells, the decision whether to fuse is likely determined pairwise by the properties of these cells. Therefore, we modeled the shapes of pairs of interacting +/+ OCLs by the arithmetic mean of their individual circularity values (MPC), and demonstrated that high MPC values are associated with pairs of OCLs that tend not to fuse. Importantly, the lack of such correlation in cultures of RQ/RQ OCLs underscores the abnormal nature of their fusion and suggests that cell shape and MPC values, per se*,* do not determine the ability of +/+ OCLs to fuse with each other. Collectively, our results indicate that SNX10 is essential for the physiological arrest of mature OCL fusion and that the R51Q mutation disrupts this regulatory function.

Results presented in this study collectively indicate that, at the cellular level, the hyperfusion phenotype of the RQ/RQ OCLs is due to loss of SNX10 function. This conclusion is supported by the significantly reduced levels of R51Q SNX10 in the mutant cells, by the ability of relatively small amounts of wild-type SNX10 protein to block formation of giant OCLs, and by the induction of hyperfusion in RAW 264.7 cells by knocking out *Snx10*. The R51Q SNX10 protein itself cannot bind PI3P or PI(3,5)P_2_, and is mislocalized in OCLs. Moreover, expression of exogenous R51Q SNX10 in RQ/RQ OCLs does not rescue their hyperfusion phenotype, indicating that the mutant SNX10 does not function like its wild-type counterpart, even when its amounts are increased in the cells. However, although these results suggest that at the molecular level, loss of function of R51Q SNX10 is caused by a combination of its severely reduced levels and by inactivity of the residual protein present in RQ/RQ OCLs, we cannot exclude at present the possibility that the R51Q SNX10 protein possesses a dominant-negative activity. Further studies are required to address this issue.

Although the exact role of SNX10 in regulating OCL fusion is still unclear, nearly all the manifestations of the R51Q mutation in this cell type, in addition to regulation of cell fusion, are related to aberrant membrane properties. These include an inability to bind specific phospholipids, decreased endocytosis, absence of ruffled borders and lack of acidification capability (Fig. 3E,F, Fig. 4D; Stein et al., 2020). Consistent with the reported role of sorting nexins in phospholipid binding and membrane trafficking (Cullen, 2008; van Weering et al., 2010), we show here that wild-type SNX10 binds PI3P and PI(3,5)P_2_, whereas R51Q SNX10 lacks this capacity (Fig. 4D), an effect that could be caused by misfolding of the mutant protein. These phospholipids are found in early and late endosomes (Haucke, 2005), in agreement with the proposed localization of SNX10 (Battaglino et al., 2019) and with the documented roles of SNX10 in regulating endocytosis and vesicular trafficking in cells (Palagano et al., 2018; Sobacchi et al., 2013). Indeed, internalization of dextran is reduced by over 40% in R51Q SNX10 OCLs compared to wild-type controls. A similar drop was noted in mononucleated RQ/RQ cells; its effect might be more limited in these less-developed cells due to their overall higher levels of uptake. It is noteworthy that inhibiting endocytosis in epidermal cells of *Caenorhabditis* *elegans* by targeting dynamin or RAB-5 results in sustained retention of the EFF-1 fusogen at the apical membrane of these cells, and promotes their excessive fusion (Smurova and Podbilewicz, 2016, 2017). Conceptually, we can envisage a similar process in mature wild-type OCLs, whereby removal of putative essential fusogens from their plasma membrane through endocytosis downregulates their fusion capacity. Defective endocytosis in mature R51Q SNX10 OCLs could result in sustained retention of such molecules in the plasma membrane and drive deregulated cell fusion. The capacity of endocytosis to affect fusion in OCLs was previously shown by Shin et al. (2014), who reported that clathrin-mediated endocytosis is required for the early-stage fusion of OCL precursor cells. The current study focuses on late-stage osteoclastogenesis, the main characteristic of which is the cessation of cell-cell fusion between mature OCLs. Therefore, it is possible that the role of endocytosis evolves to become fusion-inhibitory as OCLs mature.

Although OCL activity increases with size and nuclear number (Boissy et al., 2002; Lees and Heersche, 1999; Makris and Saffar, 1982), this study suggests that optimal OCL survival and activity are associated with a defined size range that has an upper limit, and which is actively regulated by the cell itself. The mechanistic links between the large size of RQ/RQ OCLs and their lack of ruffled border and inactivity are not clear at the present time, although they could both arise from the membranal abnormalities of these cells. Studies in which OCL hyperfusion is induced by mechanisms that do not involve SNX10 should shed further light on this issue. Nonetheless, our data suggest that the promotion of deregulated fusion that renders OCLs excessively large and fragile might enable the targeting of these cells in disease, in a manner distinct from existing strategies that inhibit formation of OCLs or induce their death.

## MATERIALS AND METHODS

### Animal studies

#### R51Q SNX10 knock-in mice

Mice carrying the R51Q mutation in SNX10 were constructed by CRISPR at The Weizmann Institute, and genotyped and maintained as described previously (Stein et al., 2020). Mice were in the C57Black/6 genetic background and were housed in a barrier facility kept at 22±2°C on a light/dark cycle of 12 h:12 h, with food and water provided *ad libitum*. Colonies were maintained by intercrossing heterozygous RQ/+ mice to produce +/+, RQ/+, and RQ/RQ littermate mice. Primary OCLs were prepared from mice of either sex, aged 4-8 weeks. All experiments were approved by the Weizmann Institute Institutional Animal Care and Use Committee and were conducted in accordance with Israeli law.

### Cell culture

#### Culture of primary mouse OCLs from spleens

Spleens from mice aged 4-8 weeks were dissociated into unsupplemented α-minimal Eagle's medium (α-MEM; Gibco-Thermo Fisher Scientific, Waltham, MA, USA or Sigma-Aldrich, St Louis, MO, USA). Following erythrocyte lysis, cells were seeded at a density of 5×10^6^ cells/well (+/+) and 2.5×10^6^ cells/well (RQ/RQ) in six-well plates, or 2×10^6^ cells/well (+/+) and 1×10^6^ cells/well (RQ/RQ) in 24-well plates. Cells were cultured in complete OCL medium [α-MEM supplemented with 10% fetal calf serum (FCS), 2 mM glutamine, 50 units/ml penicillin and 50 µg/ml streptomycin, as well as the cytokines M-CSF (20 ng/ml, Peprotech, Rehovot, Israel) and RANKL (20 ng/ml, R&D Systems, Minneapolis, MN, USA)]. Cells were grown at 37°C in 5% CO_2_ for 5-7 days with daily changes of medium. In some studies, glass coverslips were inserted into wells of 24-well plates and processed as above. For growth on bone, cells were prepared and seeded as above in wells of 24-well plates each containing 2-3 small fragments of bovine cortical bone. Cells grown on bone were cultured in complete OCL medium that included cytokines as above, with changes of medium every 48-72 h for up to 15 days. Some bone cultures were processed for scanning electron microscopy (SEM) as described previously (Stein et al., 2020).

#### Culture and manipulation of RAW264.7 cells

RAW264.7 cells, obtained from the American Type Culture Collection, were grown in Dulbecco's modified Eagle's medium (DMEM, Sigma-Aldrich), supplemented with 10% FCS, 2 mM glutamine, 50 units/ml penicillin and 50 µg/ml streptomycin. Cells were grown on plastic tissue culture plates and induced to differentiate with M-CSF and RANKL as above.

For construction of RAW LifeAct-EGFP cells, LifeAct-EGFP was isolated by PCR from pLifeAct-EGFP (Riedl et al., 2008) and cloned into the retroviral vector pBABE/Puro (Morgenstern and Land, 1990). RAW cells were transduced with retroviral particles prepared in 293 HEK cells from pBabe/Puro-LifeAct-EGFP, and selected with 5 µg/ml puromycin. For CRISPR-mediated targeting of the *Snx10* gene, candidate sgRNA sequences were selected from *Snx10* genomic sequences using the Desktop Genetics tool (deskgen.com). The sequence 5′-AAACATCTTGTGTACGAAGA-3′ was cloned into pU6-(BsaI)_CBh-Cas9-T2A-mCherry (a kind gift from Prof. Yosef Shaul, Weizmann Institute, Addgene, 135012) for expression of Cas9 and the sgRNA. This plasmid was transfected into RAW264.7 cells, using JetPEI (Polyplus Transfection, Illkirch, France), together with a plasmid that conferred resistance to blasticidin. Two days post-transfection, cells were selected with 10 µg/ml blasticidin for 24 h, and surviving cells were plated as single cells in 96-well dishes. Individual clones were genotyped by sequencing a 733 bp PCR product with the targeted site at the center, produced by 35 cycles of denaturation (95°C, 15 s), annealing (66°C, 30 s) and elongation (72°C, 30 s); primers used were 5′-GCTCGTGTGTGTTTCTCACG-3′ (forward) and 5′-AACACTTCTGGGGGCCATTC-3′ (reverse). The Synthego ICE tool (https://ice.synthego.com/) was used to deconvolute sequencing results.

### Construction and use of adenoviruses and lentiviruses to transduce OCLs

#### Adenoviruses

C-terminally FLAG-tagged wild-type and R51Q SNX10 (mouse) cDNAs were cloned into the pShuttle-CMV plasmid (Stratagene, Agilent Technologies, Inc., Santa Clara, CA, USA) and used to produce adenoviruses using the AdEasy XL adenoviral vector system (Stratagene, Agilent Technologies). Viruses were amplified using HEK293AD cells. For infection, monocytes were grown in the presence of M-CSF for 3 days, and then in M-CSF and RANKL for an additional 2 days. Then, one volume of medium from HEK293AD cells that contained adenovirus particles was added to two volumes of complete OCL growth medium volume (containing M-CSF and RANKL). Cells were incubated for 24 h, followed by incubation in fresh complete OCL medium for a further 24 h, after which cells were harvested.

#### Lentiviruses

Wild-type and R51Q SNX10 tagged at their C-terminus with FLAG-V5 were cloned into the pUltra-hot (a gift from Malcom Moore, Addgene, 24130), which also directs peptide 2A(*P*2A)-mediated independent expression of mCherry protein. Packaging was performed in HEK293 cells following transfection of the pUltra-hot plasmid and the ViraPower lentiviral packaging mix (Thermo Fisher Scientific, mix of pLP/VSVG,pLP1 and pLP2 plasmids). Medium containing lentiviral particles was collected after 48 and 72 h. For infection of RAW264.7 cells with lentiviruses, the cells were incubated with crude lentiviral-containing medium for 16 h, passaged and then fluorescence-activated cell sorted for mCherry^+^ cells using a FACSAriaIII Instrument (BD Biosciences, San Jose, CA, USA). Cells were seeded in a six-well plate (2×10^5^ cells per well). In some cases, fresh medium was added 16 h later in the presence or absence of 20 nM MG132 (Merck-Millipore, Burlington, MA, USA) for 4 h, after which cells were lysed with RIPA buffer [50 mM Tris (pH 8), 150 mM NaCl, 1% Nonidet P-40, 0.5% deoxycholate and 0.1% SDS] and analyzed by protein blotting.

### Cell analyses

#### OCL staining and immunofluorescence

For bone, OCLs grown on bone slices were stained for TRAP using a Leukocyte Acid Phosphatase kit (Sigma-Aldrich). For actin staining, cells were fixed and permeabilized by incubating the slices in 0.5% Triton X-100/3% paraformaldehyde (PFA) for 3 min, followed by 3% PFA for 20 min and three washes in PBS. Cells were then exposed to TRITC-phalloidin (Sigma-Aldrich, P1951) for 1 h at room temperature. DNA was visualized by incubating the slices with Hoechst 33342 (Molecular Probes, Eugene, OR, USA, H-3570) for 3 min.

For glass coverslips, cells were fixed and permeabilized as above, blocked in 5% horse serum and stained with Phalloidin-Alexa 488 (Cell Signaling Technology, Danvers, MA, USA, 8878S, diluted 1:2000), anti-FLAG antibody (Sigma-Aldrich, clone M2, F1804, diluted 1:200), and/or Hoechst 33342 (diluted 1:4000).

#### Live cell imaging of OCLs

Cells were cultured for 3 days in complete OCL medium supplemented with 20 ng/ml M-CSF. Cells were detached with trypsin-EDTA and seeded at 1×10^5^ cells/well on glass coverslips placed inside wells of a 24-well plate, or on µ-Slide four-well imaging chambers with a coverslip polymer bottom (Ibidi, Martinsried, Germany). Cells were cultured in complete OCL medium supplemented with 20 ng/ml M-CSF and 20 ng/ml RANKL, with daily medium changes for 3-4 days before imaging. Time-lapse images were acquired with an automated inverted microscope (DeltaVision Elite system IX71 with Resolve3D software modulus, Applied Precision, GE Healthcare, Issaquah, WA, USA) using a 10×/0.30 air objective (Olympus, Tokyo, Japan). The microscope was equipped with an environmental box kept at 37°C with a 5% CO_2_ humidified atmosphere. Images were acquired every 5 min for up to 24 h. IRM (Verschueren, 1985) time-lapse imaging was carried out using a 20×/0.7 objective at 5 min intervals between frames.

#### Dextran internalization

Splenocytes from +/+ or RQ/RQ mice were seeded on glass coverslips and differentiated into OCLs. On the day of the experiment, growth medium was replaced with OCL medium that contained 50 µM TRITC-dextran (40 kDa; Sigma-Aldrich), M-CSF and RANKL, but lacked serum. The cells were incubated for 30 min at 37°C, washed in ice-cold PBS twice, and immediately fixed in PBS containing 3.5% PFA and 2% sucrose for 15 min at room temperature. Nuclei were stained with Hoechst 33342. Cells analyzed at 0°C were initially placed on ice for 10 min before adding ice-cold medium containing TRITC-dextran, incubated for 30 min on ice and processed as above. Following mounting, the slides were analyzed using a DeltaVision Elite system IX71 (Applied Precision) using a 60×1.42 oil objective (Olympus). For each experiment, exposure times were set to visualize +/+ OCLs incubated at 37°C, and remained unchanged throughout the entire process of data collection from that experiment. The OCL SZLs, visible by actin staining, were used to define the boundary of each cell; the area of each cell and the total intensity of the TRITC signal within this area were measured using Image J. Care was taken to analyze mature round OCLs of approximately similar sizes in both genotypes.

#### Image display and analysis

Image display and analysis were performed using Fiji (Schindelin et al., 2012). For image montages, 3×3 adjacent fields with 10% overlap were stitched using the stitching plug-in (Preibisch et al., 2009). Some images were corrected for background and shading variations using the BaSic imaging tool (Peng et al., 2017). The areas and circularities of marked cells were calculated semi-automatically using Fiji software. For multinucleated cells, only cells that included three or more nuclei, and had an entire area that was within the image, were considered. For the determination of time between contact and fusion, live cell videos were run backwards and the cells were followed from fusion to the time when they first made contact. Individual mature multinucleated OCLs were followed backwards to the initial fusion event between two mononucleated cells that gave rise to them, and all intermediate fusion events were scored and analyzed. The time between first contact and separation without fusion was measured similarly starting from cells that had just separated.

For the counting of nuclei in cell images, we manually marked the cell outlines and saved them in the RoiManager. The nuclei were automatically detected from the DNA channel by enhancement using Laplacian of Gaussian filter (2 um smoothing scale), applying fixed threshold (<−0.5) and separating neighboring nuclei using watershed. To enable counting, each nucleus was shrunk to a single point. The number of nuclei in each cell was measured by counting the number of positive pixels in the single-point image within the cell border using a Fiji macro that can be obtained from the authors upon request.

#### Pit resorption studies

Cells were seeded on bone fragments as described above, and grown and analyzed as described previously (Stein et al., 2020).

#### Cell mixing studies

Splenocytes from +/+ and RQ/RQ mice were grown separately in M-CSF-containing medium for 3 days with daily medium changes. Cells were detached with trypsin/EDTA, counted and mixed in the desired proportions while maintaining the same total number of cells in each culture (1×10^5^ cells/well in 24-well plates). Cells seeded on plastic were stained for TRAP and their area measured as described above.

### qPCR analysis

RNA was extracted from spleen-derived OCLs using a NucleoSpin RNA II mini kit (Macherey-Nagel, Düren, Germany). DNase-treated RNA (1 μg) was reverse transcribed using a qScript cDNA synthesis kit (QuantaBio, Beverly, MA, USA). cDNA was subjected to qRT-PCR using SYBR FAST qPCR master mix (KAPA Biosystems, Wilmington, MA, USA) on a Step One Plus real-time PCR system (Applied Biosystems, Foster City, CA, USA). Readings were normalized to mouse actin. Primers (Table S1) were designed using Primer Express v.3 (Applied Biosystems) and validated by a standard curve and dissociation curve of the products. The fold change in target gene expression was calculated by the 2^−ΔΔCt^ relative quantification method (Applied Biosystems).

### Protein blot analysis

Cells were lysed in RIPA buffer supplemented with protease inhibitor cocktail [1 mM N-(α-aminoethyl) benzene-sulfonyl fluoride, 40 µM bestatin, 15 µM E64, 20 µM leupeptin and 15 µM pepstatin; Sigma-Aldrich]. Determination of protein concentration, SDS-PAGE and protein blotting were performed as described previously (Gil-Henn and Elson, 2003). Primary antibodies were used to detect actin (Sigma-Aldrich, clone AC-40, A3853), FLAG (Sigma-Aldrich, clone M2, F3165) and mCherry (MBL International, Des Plains, IL, USA; rabbit polyclonal, PM005). Secondary horseradish peroxidase-labeled goat-anti-mouse IgG and goat-anti-rabbit IgG antibodies were obtained from Jackson Immunoresearch Laboratories. Enhanced chemiluminescence signals were visualized using an Imagequant LAS 4000 Mini instrument (GE Healthcare Biosciences, Uppsala, Sweden) and quantified using a GelPro Analyzer V.4 (Media Cybernetics, Rockville, MD, USA).

### Targeted proteomics

Cultures of OCLs were washed once in PBS, lysed in 100 mM Tris-HCl (pH 7.4) containing 5% SDS and subjected to in-solution tryptic digestion. The resulting peptides were analyzed using nanoflow liquid chromatography columns (nanoAcquity, Waters Corp., Milford, MA, USA) coupled to high resolution high mass accuracy mass spectrometry (Q Exactive HF, Thermo Fisher Scientific). The samples were analyzed on the instrument in targeted analysis mode targeting SNX10 peptides. RQ/RQ samples were analyzed first, followed by the +/+ samples. Data were processed using Proteome Discoverer version 2.2, and searched against the mouse protein database to which a list of common lab contaminants was added. The search was performed with two search algorithms, SequestHT and Mascot. Search parameters included the following modifications: fixed modification – cysteine carbamidomethylation; variable modifications – methionine oxidation, asparagine and glutamine deamidation. A quantitative comparison was performed based on the total fragment ion intensities of SNX10 peptides after normalization to total ion current, as calculated by Skyline software (MacLean et al., 2010).

### Preparation of glutathione S-transferase fusion proteins and lipid binding assay

Wild-type or R51Q SNX10 (mouse) cDNAs, FLAG-tagged at their C-termini, were cloned into the pGEX-4T1 plasmid (Amersham/GE Healthcare). Glutathione S-transferases (GST) fusion proteins were prepared in *Escherichia* *coli* and purified as described previously (Roth et al., 2019). Proteins were quantified by blotting along with a standard concentration curve of bovine serum albumin (BSA). Lipid binding was performed using the PIP-strip membrane (P6001, Echelon Biosciences, Salt Lake City, UT, USA): membranes were blocked for 1 h at room temperature with PBS supplemented with 0.1% Triton X-100 (PBST) and 3% BSA (PBST/BSA). Following three washes in PBST, membranes were incubated for 1 h with GST proteins (0.5 µg/ml in PBST/BSA). Membranes were washed with PBST and incubated with ant-GST antibody (Sigma-Aldrich, clone GST-2, G1160) diluted 1:2000 in PBST/BSA and processed as described previously for protein blotting.

### Quantification and statistical analysis

Data were analyzed by ANOVA or by an unpaired two-tailed Student's *t*-test as indicated. Statistical analyses were performed using Prism (v. 7.04, GraphPad Software, San Diego, CA, USA) or JMP (v. 14, SAS, Cary, NC, USA). The level of statistical significance was set at *P*=0.05. Error bars are shown for data obtained from three or more biological replicates. The significant differences in cell sizes between +/+ and RQ/RQ OCLs made it impractical to blind evaluators to sample identities in most cases.

## Supplementary Material

Supplementary information

## References

[JCS254979C1] Aker, M., Rouvinski, A., Hashavia, S., Ta-Shma, A., Shaag, A., Zenvirt, S., Israel, S., Weintraub, M., Taraboulos, A., Bar-Shavit, Z. et al. (2012). An SNX10 mutation causes malignant osteopetrosis of infancy. *J. Med. Genet.* 49, 221-226. 10.1136/jmedgenet-2011-100520 22499339

[JCS254979C2] Battaglino, R. A., Jha, P., Sultana, F., Liu, W. and Morse, L. R. (2019). FKBP12: A partner of Snx10 required for vesicular trafficking in osteoclasts. *J. Cell. Biochem.* 120, 13321-13329. 10.1002/jcb.28606 30887568 PMC6570537

[JCS254979C3] Blair, H. C., Yaroslavskiy, B. B., Robinson, L. J., Mapara, M. Y., Pangrazio, A., Guo, L., Chen, K., Vezzoni, P., Tolar, J. and Orchard, P. J. (2009). Osteopetrosis with micro-lacunar resorption because of defective integrin organization. *Lab. Invest.* 89, 1007-1017. 10.1038/labinvest.2009.58 19546854 PMC2856930

[JCS254979C4] Boissy, P., Saltel, F., Bouniol, C., Jurdic, P. and Machuca-Gayet, I. (2002). Transcriptional activity of nuclei in multinucleated osteoclasts and its modulation by calcitonin. *Endocrinology* 143, 1913-1921. 10.1210/endo.143.5.8813 11956174

[JCS254979C5] Brukman, N. G., Uygur, B., Podbilewicz, B. and Chernomordik, L. V. (2019). How cells fuse. *J. Cell Biol.* 218, 1436-1451. 10.1083/jcb.201901017 30936162 PMC6504885

[JCS254979C6] Chernomordik, L. V. and Kozlov, M. M. (2008). Mechanics of membrane fusion. *Nat. Struct. Mol. Biol.* 15, 675-683. 10.1038/nsmb.1455 18596814 PMC2548310

[JCS254979C7] Chiu, Y. H. and Ritchlin, C. T. (2016). DC-STAMP: A Key Regulator in Osteoclast Differentiation. *J. Cell. Physiol.* 231, 2402-2407. 10.1002/jcp.25389 27018136 PMC4946985

[JCS254979C8] Cullen, P. J. (2008). Endosomal sorting and signalling: an emerging role for sorting nexins. *Nat. Rev. Mol. Cell Biol.* 9, 574-582. 10.1038/nrm2427 18523436

[JCS254979C9] Del Fattore, A., Cappariello, A. and Teti, A. (2008). Genetics, pathogenesis and complications of osteopetrosis. *Bone* 42, 19-29. 10.1016/j.bone.2007.08.029 17936098

[JCS254979C10] Del Fattore, A., Teti, A. and Rucci, N. (2012). Bone cells and the mechanisms of bone remodelling. *Front. Biosci. (Elite Ed)* 4, 2302-2321. 10.2741/e543 22202038

[JCS254979C11] Dworak, H. A. and Sink, H. (2002). Myoblast fusion in Drosophila. *BioEssays* 24, 591-601. 10.1002/bies.10115 12111720

[JCS254979C12] Feng, X. and Teitelbaum, S. L. (2013). Osteoclasts: New Insights. *Bone Res* 1, 11-26. 10.4248/BR201301003 26273491 PMC4472093

[JCS254979C13] Gil-Henn, H. and Elson, A. (2003). Tyrosine phosphatase-epsilon activates Src and supports the transformed phenotype of Neu-induced mammary tumor cells. *J. Biol. Chem.* 278, 15579-15586. 10.1074/jbc.M210273200 12598528

[JCS254979C14] Haucke, V. (2005). Phosphoinositide regulation of clathrin-mediated endocytosis. *Biochem. Soc. Trans.* 33, 1285-1289. 10.1042/BST0331285 16246100

[JCS254979C15] Helming, L. and Gordon, S. (2009). Molecular mediators of macrophage fusion. *Trends Cell Biol.* 19, 514-522. 10.1016/j.tcb.2009.07.005 19733078

[JCS254979C16] Hernández, J. M. and Podbilewicz, B. (2017). The hallmarks of cell-cell fusion. *Development* 144, 4481-4495. 10.1242/dev.155523 29254991

[JCS254979C17] Itzstein, C., Coxon, F. P. and Rogers, M. J. (2011). The regulation of osteoclast function and bone resorption by small GTPases. *Small GTPases* 2, 117-130. 10.4161/sgtp.2.3.16453 21776413 PMC3136942

[JCS254979C18] Jansen, I. D., Vermeer, J. A. F., Bloemen, V., Stap, J. and Everts, V. (2012). Osteoclast fusion and fission. *Calcif. Tissue Int.* 90, 515-522. 10.1007/s00223-012-9600-y 22527205 PMC3349023

[JCS254979C19] Lees, R. L. and Heersche, J. N. (1999). Macrophage colony stimulating factor increases bone resorption in dispersed osteoclast cultures by increasing osteoclast size. *J. Bone Miner. Res.* 14, 937-945. 10.1359/jbmr.1999.14.6.937 10352102

[JCS254979C20] Levaot, N., Ottolenghi, A., Mann, M., Guterman-Ram, G., Kam, Z. and Geiger, B. (2015). Osteoclast fusion is initiated by a small subset of RANKL-stimulated monocyte progenitors, which can fuse to RANKL-unstimulated progenitors. *Bone* 79, 21-28. 10.1016/j.bone.2015.05.021 26008608

[JCS254979C21] MacLean, B., Tomazela, D. M., Shulman, N., Chambers, M., Finney, G. L., Frewen, B., Kern, R., Tabb, D. L., Liebler, D. C. and MacCoss, M. J. (2010). Skyline: an open source document editor for creating and analyzing targeted proteomics experiments. *Bioinformatics* 26, 966-968, 10.1093/bioinformatics/btq054. 20147306 PMC2844992

[JCS254979C22] Makris, G. P. and Saffar, J. L. (1982). Quantitative relationship between osteoclasts, osteoclast nuclei and the extent of the resorbing surface in hamster periodontal disease. *Arch. Oral Biol.* 27, 965-969. 10.1016/0003-9969(82)90104-2 6961912

[JCS254979C23] Martens, S. and McMahon, H. T. (2008). Mechanisms of membrane fusion: disparate players and common principles. *Nat. Rev. Mol. Cell Biol.* 9, 543-556. 10.1038/nrm2417 18496517

[JCS254979C24] Morgenstern, J. P. and Land, H. (1990). Advanced mammalian gene transfer: high titre retroviral vectors with multiple drug selection markers and a complementary helper-free packaging cell line. *Nucleic Acids Res.* 18, 3587-3596. 10.1093/nar/18.12.3587 2194165 PMC331014

[JCS254979C25] Nakamura, I., Duong le, T., Rodan, S. B. and Rodan, G. A. (2007). Involvement of alpha(v)beta3 integrins in osteoclast function. *J. Bone Miner. Metab.* 25, 337-344. 10.1007/s00774-007-0773-9 17968485

[JCS254979C26] Novack, D. V. and Teitelbaum, S. L. (2008). The osteoclast: friend or foe? *Annu. Rev. Pathol.* 3, 457-484. 10.1146/annurev.pathmechdis.3.121806.151431 18039135

[JCS254979C27] Oren-Suissa, M. and Podbilewicz, B. (2007). Cell fusion during development. *Trends Cell Biol.* 17, 537-546. 10.1016/j.tcb.2007.09.004 17981036

[JCS254979C28] Palagano, E., Menale, C., Sobacchi, C. and Villa, A. (2018). Genetics of osteopetrosis. *Curr. Osteoporos Rep.* 16, 13-25. 10.1007/s11914-018-0415-2 29335834

[JCS254979C29] Pangrazio, A., Fasth, A., Sbardellati, A., Orchard, P. J., Kasow, K. A., Raza, J., Albayrak, C., Albayrak, D., Vanakker, O. M., De Moerloose, B. et al. (2013). SNX10 mutations define a subgroup of human autosomal recessive osteopetrosis with variable clinical severity. *J. Bone Miner. Res.* 28, 1041-1049. 10.1002/jbmr.1849 23280965

[JCS254979C30] Peng, T., Thorn, K., Schroeder, T., Wang, L., Theis, F. J., Marr, C. and Navab, N. (2017). A BaSiC tool for background and shading correction of optical microscopy images. *Nat. Commun.* 8, 14836. 10.1038/ncomms14836 28594001 PMC5472168

[JCS254979C31] Preibisch, S., Saalfeld, S. and Tomancak, P. (2009). Globally optimal stitching of tiled 3D microscopic image acquisitions. *Bioinformatics* 25, 1463-1465. 10.1093/bioinformatics/btp184 19346324 PMC2682522

[JCS254979C32] Pustylnikov, S., Sagar, D., Jain, P. and Khan, Z. K. (2014). Targeting the C-type Lectins-Mediated Host-Pathogen Interactions with Dextran. *J. Pharm. Pharm. Sci.* 17, 371-392. 10.18433/J3N590 25224349 PMC5553543

[JCS254979C33] Riedl, J., Crevenna, A. H., Kessenbrock, K., Yu, J. H., Neukirchen, D., Bista, M., Bradke, F., Jenne, D., Holak, T. A., Werb, Z. et al. (2008). Lifeact: a versatile marker to visualize F-actin. *Nat. Methods* 5, 605-607. 10.1038/nmeth.1220 18536722 PMC2814344

[JCS254979C34] Rodan, S. B. and Rodan, G. A. (1997). Integrin function in osteoclasts. *J. Endocrinol.* 154 Suppl, S47-S56. 9379137

[JCS254979C35] Roth, L., Wakim, J., Wasserman, E., Shalev, M., Arman, E., Stein, M., Brumfeld, V., Sagum, C. A., Bedford, M. T., Tuckermann, J. et al. (2019). Phosphorylation of the phosphatase PTPROt at Tyr(399) is a molecular switch that controls osteoclast activity and bone mass in vivo. *Sci. Signal.* 12, eaau0240. 10.1126/scisignal.aau0240 30622194

[JCS254979C36] Schejter, E. D. (2016). Myoblast fusion: Experimental systems and cellular mechanisms. *Semin. Cell Dev. Biol.* 60, 112-120. 10.1016/j.semcdb.2016.07.016 27423913

[JCS254979C37] Schindelin, J., Arganda-Carreras, I., Frise, E., Kaynig, V., Longair, M., Pietzsch, T., Preibisch, S., Rueden, C., Saalfeld, S., Schmid, B. et al. (2012). Fiji: an open-source platform for biological-image analysis. *Nat. Methods* 9, 676-682. 10.1038/nmeth.2019 22743772 PMC3855844

[JCS254979C38] Shin, N. Y., Choi, H., Neff, L., Wu, Y., Saito, H., Ferguson, S. M., De Camilli, P. and Baron, R. (2014). Dynamin and endocytosis are required for the fusion of osteoclasts and myoblasts. *J. Cell Biol.* 207, 73-89. 10.1083/jcb.201401137 25287300 PMC4195819

[JCS254979C39] Smurova, K. and Podbilewicz, B. (2016). RAB-5- and DYNAMIN-1-mediated endocytosis of EFF-1 fusogen controls cell-cell fusion. *Cell Rep* 14, 1517-1527. 10.1016/j.celrep.2016.01.027 26854231 PMC4761113

[JCS254979C40] Smurova, K. and Podbilewicz, B. (2017). Endocytosis regulates membrane localization and function of the fusogen EFF-1. *Small GTPases* 8, 177-180. 10.1080/21541248.2016.1211399 27470417 PMC5584736

[JCS254979C41] Sobacchi, C., Schulz, A., Coxon, F. P., Villa, A. and Helfrich, M. H. (2013). Osteopetrosis: genetics, treatment and new insights into osteoclast function. *Nat. Rev. Endocrinol.* 9, 522-536. 10.1038/nrendo.2013.137 23877423

[JCS254979C42] Soe, K., Hobolt-Pedersen, A. S. and Delaisse, J. M. (2015). The elementary fusion modalities of osteoclasts. *Bone* 73, 181-189. 10.1016/j.bone.2014.12.010 25527420

[JCS254979C43] Stattin, E. L., Henning, P., Klar, J., McDermott, E., Stecksen-Blicks, C., Sandstrom, P. E., Kellgren, T. G., Ryden, P., Hallmans, G., Lonnerholm, T. et al. (2017). SNX10 gene mutation leading to osteopetrosis with dysfunctional osteoclasts. *Sci. Rep.* 7, 3012. 10.1038/s41598-017-02533-2 28592808 PMC5462793

[JCS254979C44] Stein, M., Barnea-Zohar, M., Shalev, M., Arman, E., Brenner, O., Winograd-Katz, S., Gerstung, J., Thalji, F., Kanaan, M., Elinav, H. et al. (2020). Massive osteopetrosis caused by non-functional osteoclasts in R51Q SNX10 mutant mice. *Bone* 136, 115360. 10.1016/j.bone.2020.115360 32278070

[JCS254979C45] Teasdale, R. D. and Collins, B. M. (2012). Insights into the PX (phox-homology) domain and SNX (sorting nexin) protein families: structures, functions and roles in disease. *Biochem. J.* 441, 39-59. 10.1042/BJ20111226 22168438

[JCS254979C46] Teitelbaum, S. L. (2007). Osteoclasts: what do they do and how do they do it? *Am. J. Pathol.* 170, 427-435. 10.2353/ajpath.2007.060834 17255310 PMC1851862

[JCS254979C47] Teitelbaum, S. L. (2011). The osteoclast and its unique cytoskeleton. *Ann. N. Y. Acad. Sci.* 1240, 14-17. 10.1111/j.1749-6632.2011.06283.x 22172034

[JCS254979C48] van Weering, J. R., Verkade, P. and Cullen, P. J. (2010). SNX-BAR proteins in phosphoinositide-mediated, tubular-based endosomal sorting. *Semin. Cell Dev. Biol.* 21, 371-380. 10.1016/j.semcdb.2009.11.009 19914387 PMC4052211

[JCS254979C49] Verschueren, H. (1985). Interference reflection microscopy in cell biology: methodology and applications. *J. Cell Sci.* 75, 279-301. 3900106 10.1242/jcs.75.1.279

[JCS254979C50] Willkomm, L. and Bloch, W. (2015). State of the art in cell-cell fusion. *Methods Mol. Biol.* 1313, 1-19. 10.1007/978-1-4939-2703-6_1 25947653

[JCS254979C51] Witwicka, H., Hwang, S. Y., Reyes-Gutierrez, P., Jia, H., Odgren, P. E., Donahue, L. R., Birnbaum, M. J. and Odgren, P. R. (2015). Studies of OC-STAMP in osteoclast fusion: a new knockout mouse model, rescue of cell fusion, and transmembrane topology. *PLoS ONE* 10, e0128275. 10.1371/journal.pone.0128275 26042409 PMC4456411

[JCS254979C52] Worby, C. A. and Dixon, J. E. (2002). Sorting out the cellular functions of sorting nexins. *Nat. Rev. Mol. Cell Biol.* 3, 919-931. 10.1038/nrm974 12461558

[JCS254979C53] Ye, L., Morse, L. R., Zhang, L., Sasaki, H., Mills, J. C., Odgren, P. R., Sibbel, G., Stanley, J. R., Wong, G., Zamarioli, A. et al. (2015). Osteopetrorickets due to Snx10 deficiency in mice results from both failed osteoclast activity and loss of gastric acid-dependent calcium absorption. *PLoS Genet.* 11, e1005057. 10.1371/journal.pgen.1005057 25811986 PMC4374855

[JCS254979C54] Zhou, C., Wang, Y., Peng, J., Li, C., Liu, P. and Shen, X. (2017). SNX10 plays a critical role in MMP9 secretion via JNK-p38-ERK signaling pathway. *J. Cell. Biochem.* 118, 4664-4671. 10.1002/jcb.26132 28498635

[JCS254979C55] Zhu, C. H., Morse, L. R. and Battaglino, R. A. (2012). SNX10 is required for osteoclast formation and resorption activity. *J. Cell. Biochem.* 113, 1608-1615. 22174188 10.1002/jcb.24029

