# Supplementary material for: An SNX10-dependent mechanism downregulates fusion between mature osteoclasts
Source: J Cell Sci. 2021 May 11;134(9):jcs254979. doi: 10.1242/jcs.254979 (PMC8182410; doi:10.1242/jcs.254979)
Supplement: Supplementary information [file joces-134-254979-s1.pdf]

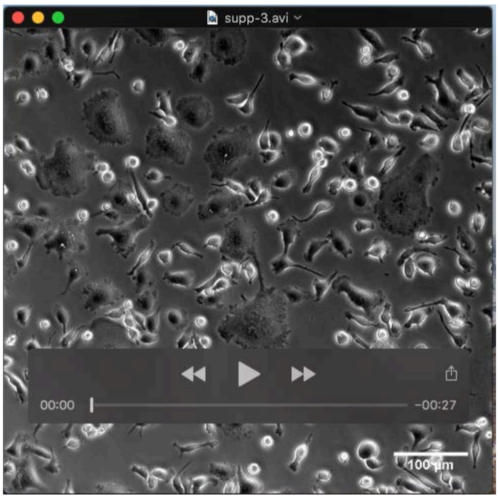

Movie 1: Phase light microscopy live cell imaging of +/+ monocytes as they fuse into OCLs. Individual images were obtained at 5-minute intervals for a period of 21 hours. Note maturation of three adjacent multi-nucleated cells at bottom left of screen between t=450-755 minutes, and lack of fusion between pairs of mature, round OCLs. Still images from this movie are presented in Figures 2C, 2D.

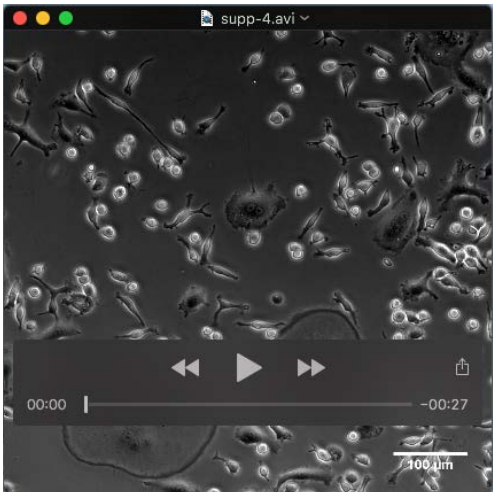

Movie 2: Phase light microscopy live cell imaging of RQ/RQ monocytes as they fuse into OCLs. Individual images were obtained at 5-minute intervals for a period of 21 hours. Note extensive and continuous fusion between mature OCLs. Still images from this movie are presented in Figures 2C, 2D.

Journal of Cell Science • Supplementary information

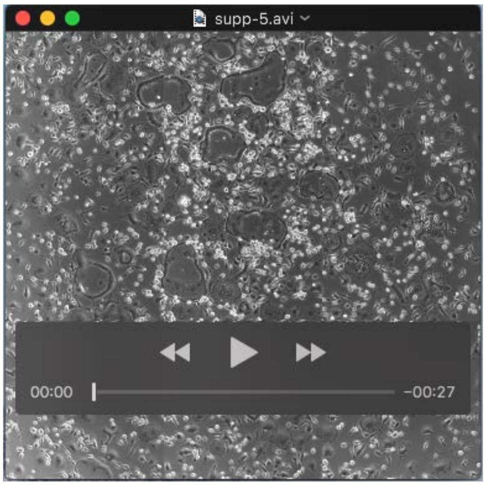

Movie 3: Phase light microscopy live cell imaging of +/+ monocytes as they fuse into OCLs. Image is a montage of 3X3 fields and covers an area of 2 X 2 millimeters. Individual images were obtained at 5-minute intervals for a period of 21 hours. Note lack of significant fusion between adjacent large, mature OCLs at center-top during the last third of the movie.

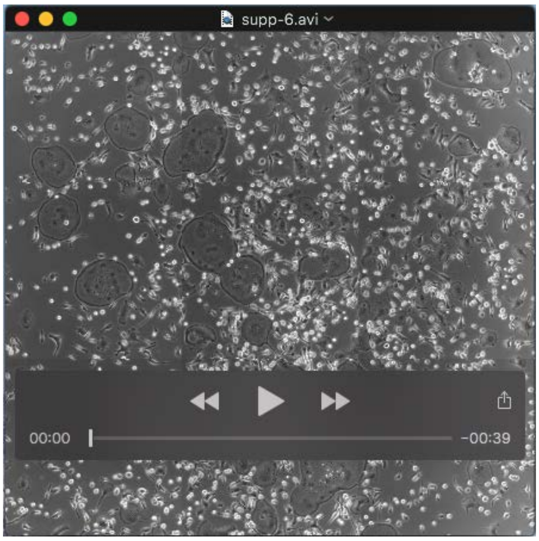

Movie 4: Phase light microscopy live cell imaging of RQ/RQ monocytes as they fuse into a single OCL. Image is a montage of 3X3 fields and covers an area of 2 X 2 millimeters. Individual images were obtained at 5-minute intervals for a period of 21 hours. At the end of this movie, most of the field of view is covered by part of a single cell.

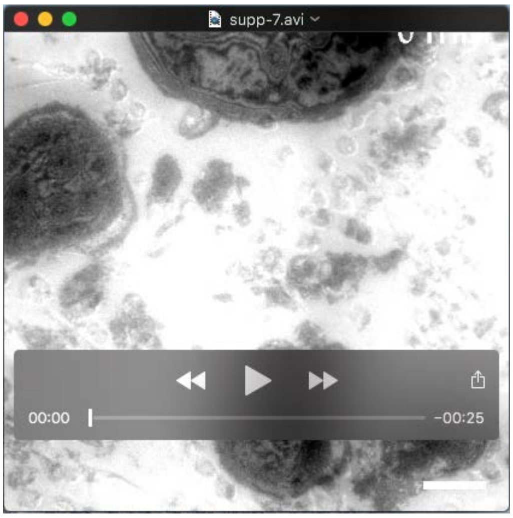

Movie 5: Interference reflection microscopy live cell imaging of +/+ monocytes and OCLs as they interact in culture. All fusion events involve at least one small, immature cell. Images from this movie are shown in Figure 3A and Figure S2.

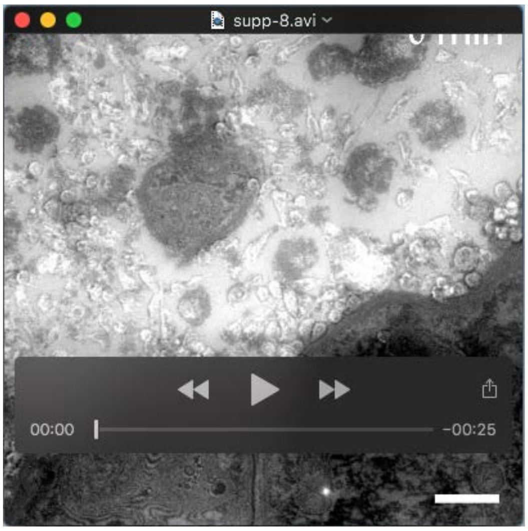

Movie 6: Interference reflection microscopy live cell imaging of RQ/RQ monocytes and OCLs as they interact and fuse in culture. Note extensive fusion events between large OCLs. Images from this movie are shown in Figure 3A and Figure S3.

Journal of Cell Science • Supplementary information

Table S1

Table S1: List of qPCR primers used in this study. Primers were generated and validated as detailed in the Methods section.

Protein	Gene	Accession number	FW	Rev
TRAP	*Acp5*	NM_007388.3	GAT GCC AGC GAC AAG AGG TT	CAT ACC AGG GGA TGT TGC GAA
βActin	*Actb*	NM_007393.5	CTA AGG CCA ACC GTG AAA AG	GGG GTG TTG AAG GTC TCA AA
ATP6V0D2	*Atp6v0d2*	NM_175406.3	GTG AGA CCT TGG AAG ACC TGA AA	TCC TCA TCT CCG TGT CAA TTT TG
CLCN7	*Clcn7*	NM_011930.4	AAG CGA CAC AGC GTC TAA TCA C	TCC ACA AAC ACC TTA TGC TTC AA
Cathepsin K	*Ctsk*	NM_007802.4	GGC CAG GAT GAA AGT TGT ATG TAT AA	TCT CGT TCC CCA CAG GAA TC
DC-STAMP	*Dcstamp*	NM_029422.4	TCC TCC ATG AAC AAA CAG TTC CA	AGA CGT GGT TTA GGA ATG CAG CTC
NFATc1	*Nfatc1*	NM_016791.4	CCT GGA GAT CCC GTT GCT T	CGA TGT CTG TCT CCC CTT TCC
OC-STAMP	*Ocstamp*	NM_029021.1	TCA CAG TCA AAT ATG ACG CCT CAT	GGT GGT TGA GCC TGT GGT AGA
SNX-10	*Snx10*	NM_028035.4	ACG CGT TGC TGG TAC AAT TAC C	AAG AGG TGG AGG CTG CTA TCG
RANK	*Tnfrsf11a*	NM_009399.3	CAG GAG AGG CAT TAT GAG CA	GGT ACT TTC CTG GTT CGC AT

Supplementary Figures

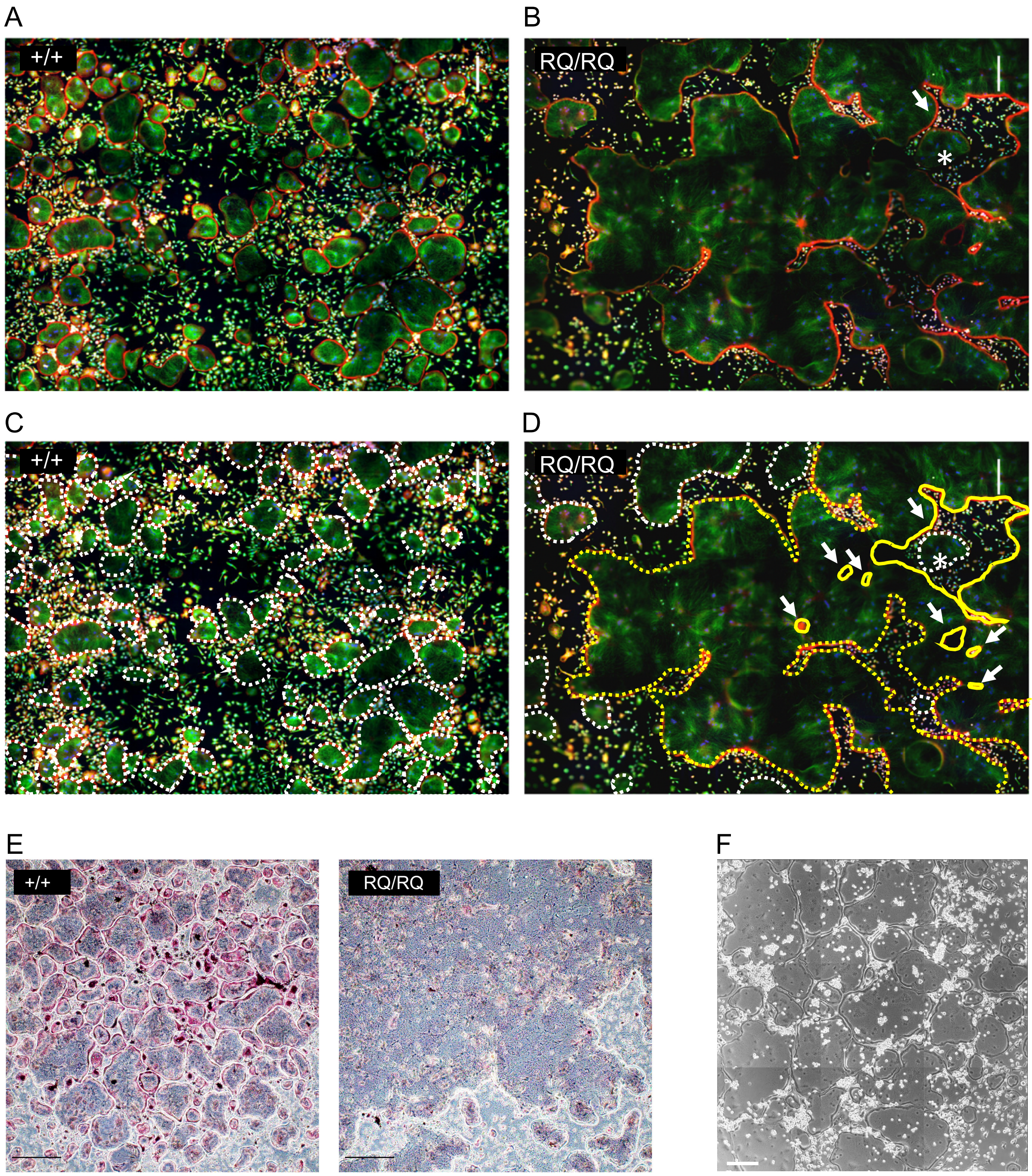

Figure S1: (A-D) Demarcation of individual OCLs in Figures 1A, 1B. **(A,B)**. Cultures of +/+ and RQ/RQ OCLs, stained for actin (red), tubulin (green), and DNA (blue) as shown in Figure 1. **(C, D).** Same images highlighting mature, round OCLs (dashed lines). Dashed yellow line (D) highlights part of a single large OCL in the RQ/RQ image. Solid yellow lines (D) denote areas located outside the cell that are completely surrounded by the cell. These areas can be sufficiently large to host smaller mature OCLs (asterisk) and gradually grow smaller (e.g., arrows) until they are completely absorbed by the cell. Scale bars (upper right): 200 µm. **(E) Large OCLs produced from bone marrow cells of +/+ and RQ/RQ mice.** Cells were differentiated with M-CSF and RANKL, and stained for tartarate-resistant acid phosphatase (TRAP). Scale bars: 500 µm. **(F) Mature, round +/+ OCLs that are surrounded by similarly mature OCLs tend not to fuse with each other.** Phase microscopy image is from the end of Movie 3 (t=1345'). The cluster of large OCLs at center-top of this image was present and stable during the 4 hours that preceded this image. Bar: 200 µm.

Journal of Cell Science • Supplementary information

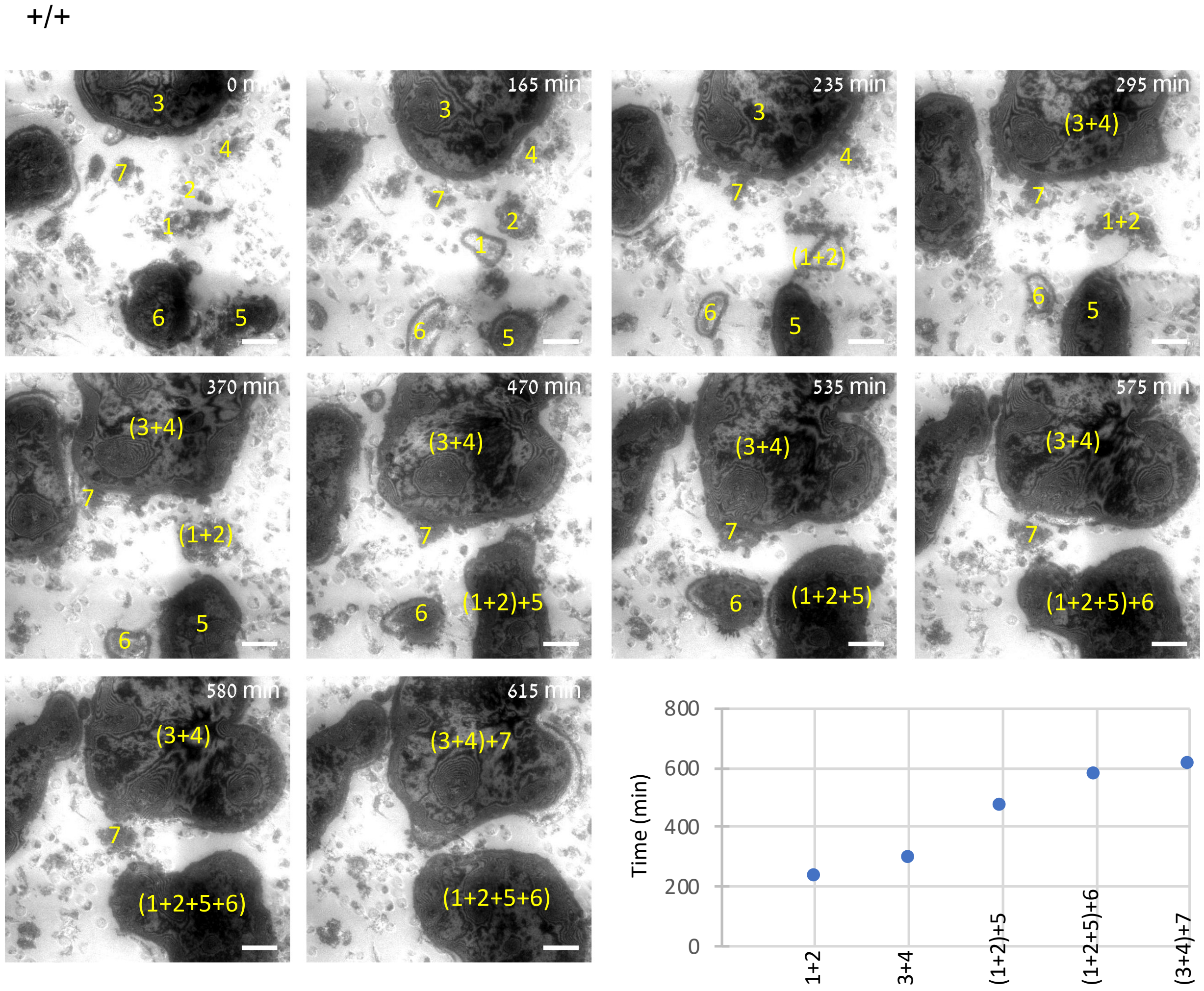

Figure S2 Images captured from Movie 5, showing interference reflection microscopy (IRM) live cell imaging of +/+ monocytes and OCLs are they interact and fuse in culture. Individual cells are marked by numbers; parentheses indicate cells that have fused. Bottom left: graph depicting time course of fusion events shown in this figure. Note that all fusion events involve at least one small, immature OCL. Bar: 100 µm.

Journal of Cell Science • Supplementary information

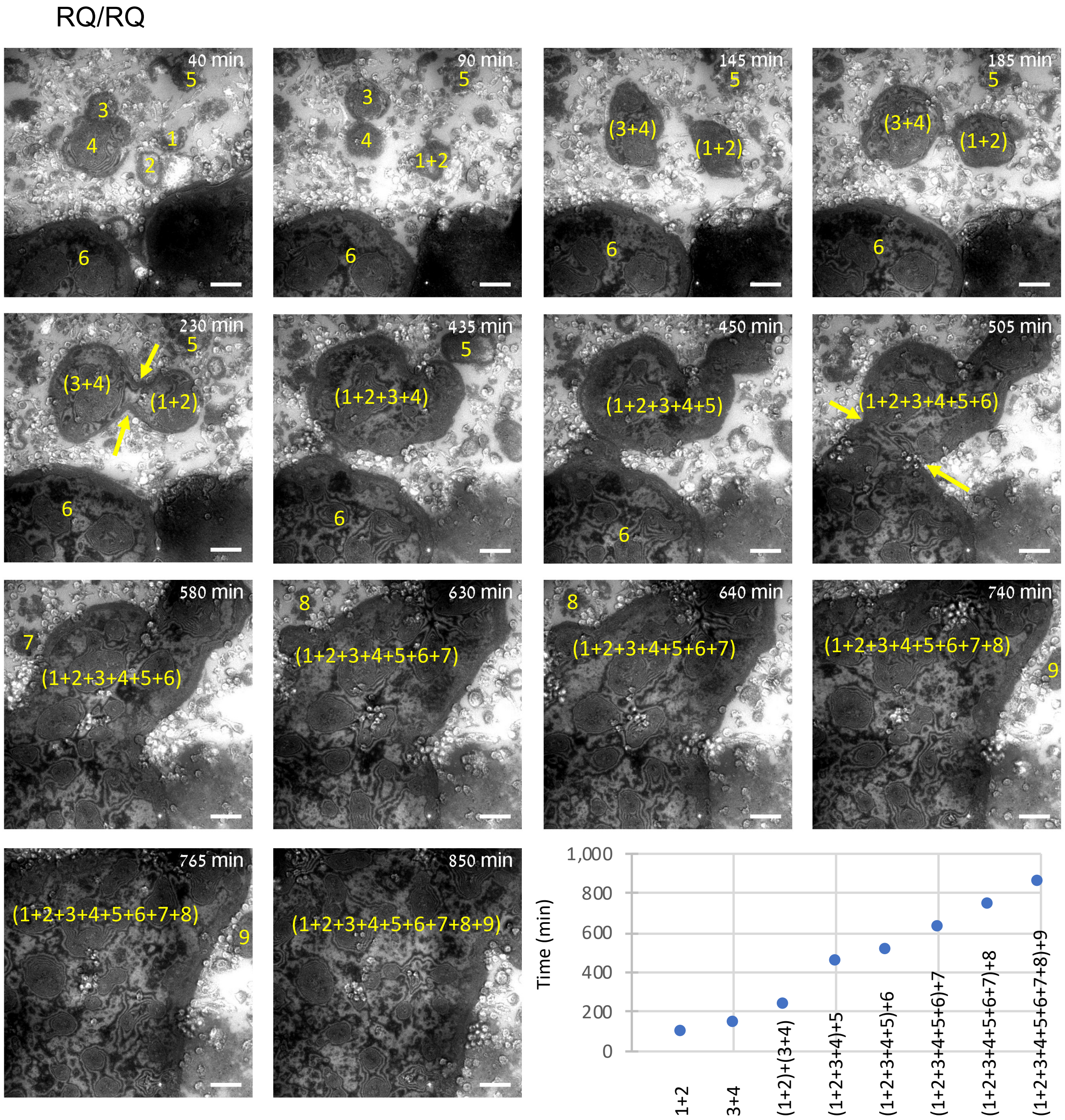

Figure S3: Images captured from Movie 6, showing IRM live cell imaging of RQ/RQ monocytes and OCLs are they interact and fuse in culture. Individual cells are marked by numbers; parentheses indicate cells that have fused. Bottom left: graph depicting time course of fusion events shown in this figure. Note multiple fusion events between pairs of large, mature OCLs. Also note appearance of lightly-colored area at the junction of cells as they fuse (arrows, t=505 min), indicating short-term separation of the fusion area from the underlying surface. Bar: 100 µm.

Journal of Cell Science • Supplementary information

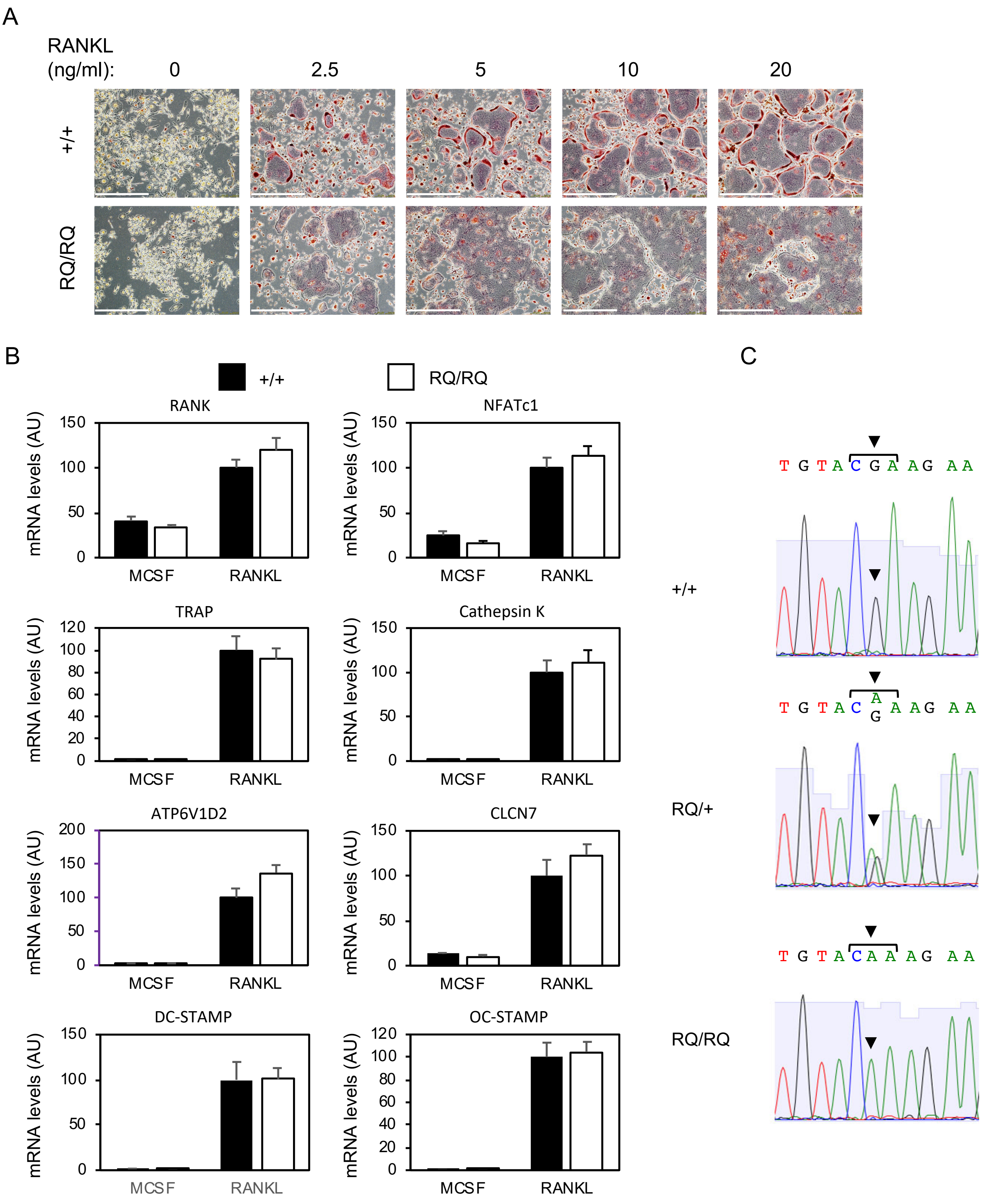

Figure S4: (A) Increased fusion of RQ/RQ OCLs is not due to hypersensitivity to RANKL. Representative images of TRAP-stained splenocytes from +/+ and RQ/RQ mice after 5 days growth in different concentrations of RANKL as indicated. Bars: 250 mm. See also Figure 3D. Data is from one experiment, representative of 3 performed. **(B) mRNA levels of key osteoclast-related genes in +/+ and RQ/RQ OCLs.** mRNA samples from +/+ and RQ/RQ splenocytes that were grown with M-CSF alone

Journal of Cell Science • Supplementary information

(MCSF) or with M-CSF and RANKL (RANKL) for 5 days were reverse-transcribed and analyzed by qPCR. Data are mean±SD from N=12 +/+ and N=14 RQ/RQ mice. Data were analyzed by one-way ANOVA followed by a Tukey-Kramer post-hoc for multiple comparisons test; in all cases, the differences between the cytokines were significant, but the differences between genotypes treated with the same cytokine were non-significant. **(C) Expression of *Snx10* mRNA in +/+ , RQ/+, and RQ/RQ OCLs.** Total RNA from OCLs of the indicated genotypes was reverse-transcribed, amplified by PCR, and sequenced. +/+ and RQ/RQ samples express either +/+ or mutant mRNAs, respectively, while the heterozygous (RQ/+) sample expresses both. Sequence shown is in the sense orientation. The mutated triplet is marked.

Journal of Cell Science • Supplementary information

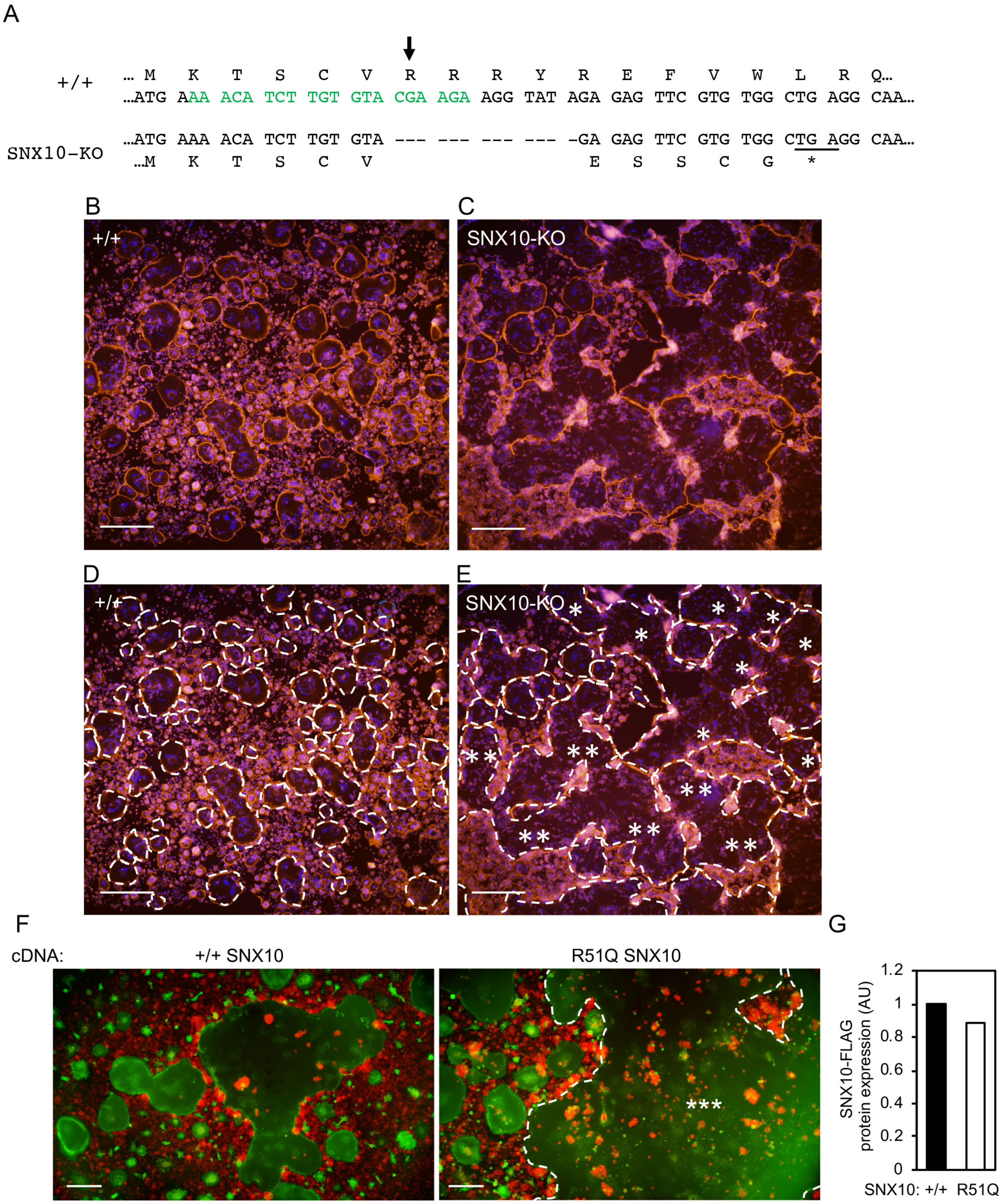

Figure S5: SNX10-knockout RAW264.7 cells were produced by CRISPR. **(A)** Shown are the DNA and translated protein sequences of the +/+ *Snx10* allele (top) and of the targeted allele, which carries a 13bp deletion (bottom). No other allele was detected by DNA sequencing, indicating that this clone is either homozygous for this deletion of that the second allele carries a massive deletion that prevents

Journal of Cell Science • Supplementary information

its amplification by PCR. R51 is marked by an arrow and the sgRNA used is shown in green. (**B-E**) Demarcation of individual OCLs in Figure 5A. (**B, C**). Cultures of differentiated +/+ (wild-type) and SNX10-KO RAW 264.7 OCLs, stained for actin (red) and DNA (blue), as shown in Figure 5A. (**D, E).** Same images highlighting mature, round OCLs (dashed lines). Panel E contains two large OCLs, whose areas are marked by one or two asterisks, respectively. Scale bars: 500 µm. **(F) R51Q SNX10 cannot rescue the hyper-fusion phenotype of RQ/RQ OCLs.** Cells used in this study were primary splenocytes from RQ/RQ mice carrying the mT/mG transgene (Muzumdar et al., 2007)**,** which ubiquitously expresses Tomato that switches to EGFP following Cre-mediated recombination. The cells also express Cre under the direction of the cathepsin-K promoter (Cath-K-Cre), hence mononuclear cells are shown in red and maturing OCLs in green. FLAG-tagged +/+ and R51Q SNX10 were expressed in the cells using adenoviruses. Care was taken to express similar amounts of exogenous +/+ and R51Q SNX10 protein in these cultures since the R51Q SNX10 protein is unstable in OCLs (Figures 4B,C). +/+ SNX10 significantly reduced the hyper-fusion phenotype of the cells (left), while similar levels of R51Q SNX10 did not (right; dashed line and asterisk triplet mark the cytosol of a single OCL, which extends significantly beyond the field of view). **(G)** Relative protein expression of FLAG-SNX10 in both cultures from the experiment shown in (F), as determined by protein blotting with anti-FLAG antibodies. Scale bars: 200 µm.

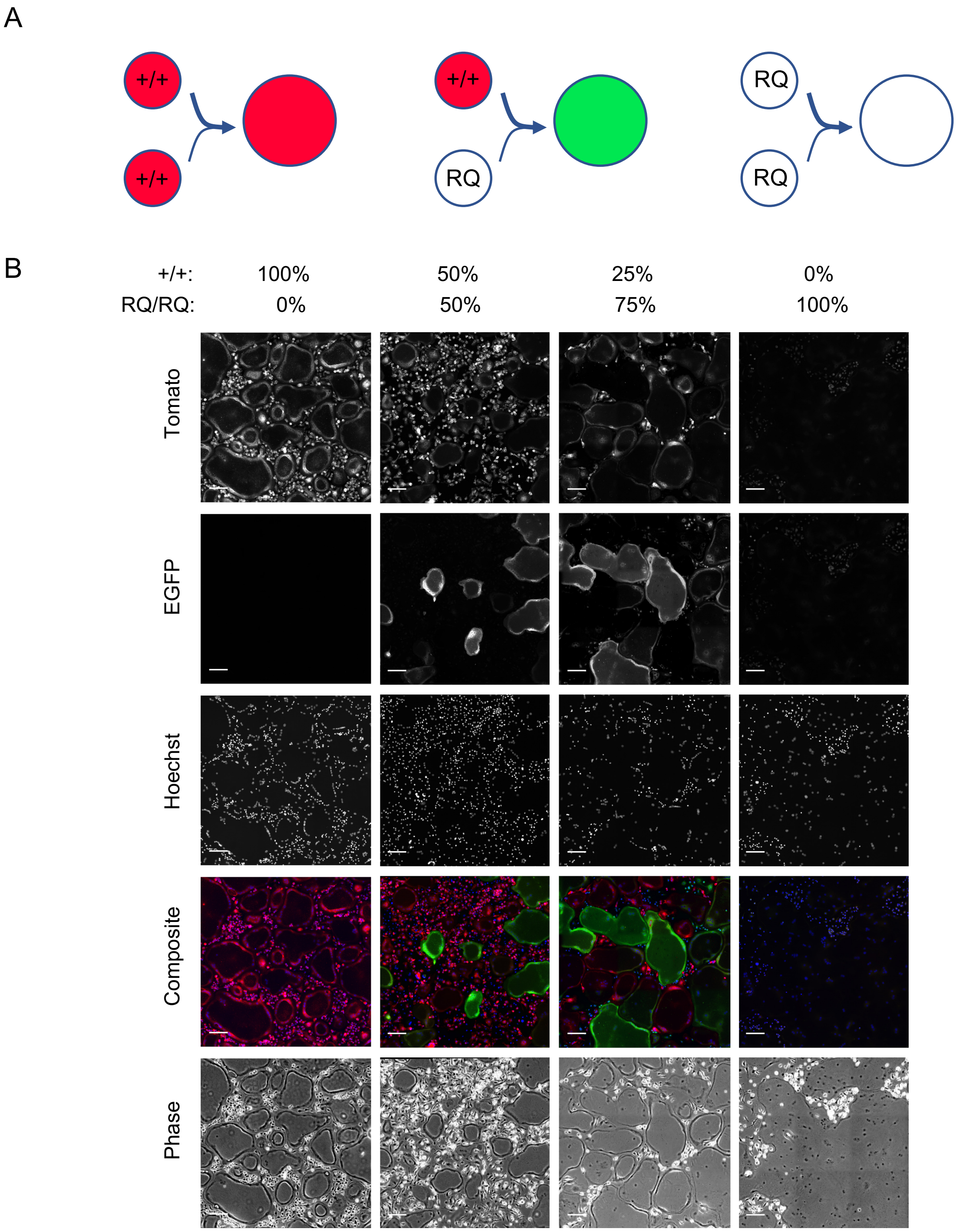

Figure S6: +/+ and RQ/RQ OCLs can fuse with each other to form genetically hybrid heterokaryons. Cells used in this study were primary splenocytes from +/+ mT/mG mice, described in Figure S5F, which express Tomato and switch to EGFP in the presence of Cre, and from RQ/RQ mice carrying the Cathepsin-K Cre transgene, which are colorless. Fusion between both genotypes in culture induces the Tomato-EGFP switch. **(A)** Scheme showing the possible outcomes of fusion in this system. **(B)** Cells were grown separately for three days in the presence of M-CSF, counted, mixed in the indicated proportions, and grown in the presence of M-CSF and RANKL for 5 additional days. Note that the EGFP signal is present only in the mixed cultures. Bars: 100 µm. Images are representative of two experiments.

Journal of Cell Science • Supplementary information

Supplementary references:

Muzumdar, M. D., Tasic, B., Miyamichi, K., Li, L. and Luo, L. (2007). A global double-fluorescent Cre reporter mouse. *Genesis* **45**, 593-605.

Journal of Cell Science • Supplementary information
